# Recent Advances in the Optimization of Nucleic Acid Aptamers and Aptasensors

**DOI:** 10.3390/bios15100641

**Published:** 2025-09-25

**Authors:** Yuan Wang, Mengyan Nie

**Affiliations:** Institute for Materials Discovery, University College London, Malet Place, London WC1E 7JE, UK; zczqywa@ucl.ac.uk

**Keywords:** nucleic acid aptamers, systematic evolution of ligands by exponential enrichment (SELEX), Aptasensors, post-SELEX optimization, aptamer truncation, predictive algorithms

## Abstract

Nucleic acid aptamers are single-stranded DNA or RNA molecules that can bind to a target with high specificity and affinity, as screened by the Systematic Evolution of Ligands by Exponential Enrichment (SELEX). In recent years, SELEX technologies have been significantly advanced for the screening of aptamers for a variety of target molecules, cells, and even bacteria and viruses. By integrating recent advances of emerging technologies with SELEX, novel screening technologies for nucleic acid aptamers have emerged with improved screening efficiency, reduced production costs and enhanced aptamer performance for a wide range of applications in medical diagnostics, drug delivery, and environmental monitoring. Aptasensors utilize aptamers to detect a wide range of analytes, allowing for the accurate identification and determination of small molecules, proteins, and even whole cells with remarkable specificity and sensitivity. Further optimization of the aptasensor can be achieved by aptamer truncation, which not only maintains the high specificity and affinity of the aptamer binding with the target analytes, but also reduces the manufacturing cost. Predictive models also demonstrate the powerful capability of determination of the minimal functional sequences by simulation of aptamer–target interaction processes, thus effectively shortening the aptamer screening procedure and reducing the production costs. This paper summarizes the research progress of protein-targeted aptamer screening in recent years, introduces several typical aptasensors at present, discusses the optimization methods of aptasensors by combining efficient SELEX with advanced predictive algorithms or post-SELEX processes, as well as the challenges and opportunities faced by aptasensors.

## 1. Introduction

Aptamers are typically short, single-stranded DNA or RNA oligonucleotides that bind to specific targets which include metal ions, small molecules, peptides, and even proteins expressed on the surface of bacteria, virus, or human cells and are difficult to obtain high-affinity antibodies [1,2,3]. This unique property has turned aptamers into versatile tools for target molecule identification and detection in biological samples, playing an important role in synthetic biology and healthcare industries [4,5,6,7,8,9,10,11]. While having a similar binding affinity and specificity to antibodies, nucleic acid aptamers can be chemically synthesized and demonstrate many advantages over antibodies, such as easy chemical modification, better stability, and low production costs. Moreover, due to their smaller size (typically 20–80 nucleotides in length or 10–20 kDa in weight), aptamers have better tissue permeability, are easily accessible to cells, and are faster to bind with their targets. As our body naturally has nucleic acid circulating, synthetic aptamers are usually not recognized as foreign by the immune system, thus do not elicit an immune response from cells [12]. Aptamers also have the ability to flexibly fold to recognize the target molecules which may be large or small in the biological samples and need to be bonded with the aptamer sensing elements of biosensors [13,14]. In addition, aptamers are easy to preserve and can be kept in a suitable environment for a long time, which makes the application of aptamers as molecular recognition elements in bioassays suitable. Table 1 summarizes the advantages and limitations of aptamers compared with antibodies.

**Figure 1:**
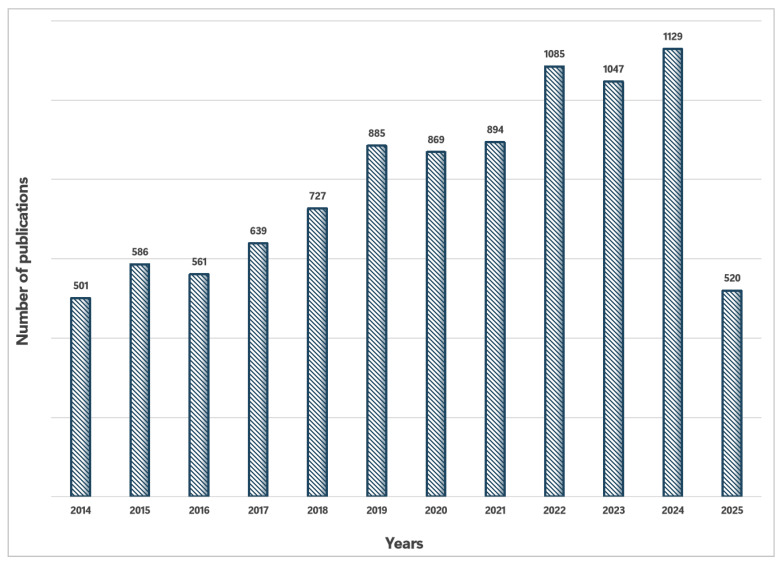
The number of published research papers on aptamers or aptasensors each year in the past decade.

As there are many advantages of using aptamers over antibodies, aptamers have been extensively investigated for biosensors. As shown in Figure 1, the publications found by searching PubMed with the keywords of either aptamer or aptasensor have increased every year from 2014 up to 20 May 2025.

## 2. Nucleic Acid Aptamer

### 2.1. Nucleic Acid Aptamer

As aptamers are short, single-stranded DNA or RNA molecules, they can change shapes dramatically. The binding of the nucleic acid aptamer to the target molecule is achieved through its unique three-dimensional conformation, which is characterized by the shape of hairpins, inner loops, pseudoknots, bulges, or G-quadruplexes [15]. Binding between the nucleic acid aptamer and the target molecule occurs through van der Waals forces, hydrogen bonding, and electrostatic forces [16,17]. When a nucleic acid aptamer binds with a target molecule, if the target molecule is small, the nucleic acid aptamer will cover the target molecule and wrap around the surface of its molecule through its unique helical structure. If the target molecule is large, the nucleic acid aptamer will form adaptive-like structures in the clefts and gaps on the surface of the large molecule. Nucleic acid aptamers have a good folding ability, which allows the nucleic acid aptamer to fully adapt to the size of the target molecule [18]. Nucleic acid aptamers can fully bind to target molecules and adapt to numerous target molecules of different shapes. The targets of nucleic acid aptamers include, but are not limited to, peptides [19], proteins [20,21], small organic molecules [22], various metal ions [23], and even molecules on the surface of bacteria [24], viruses [25], or human cells [26].

### 2.2. SELEX Technology

**Figure 2:**
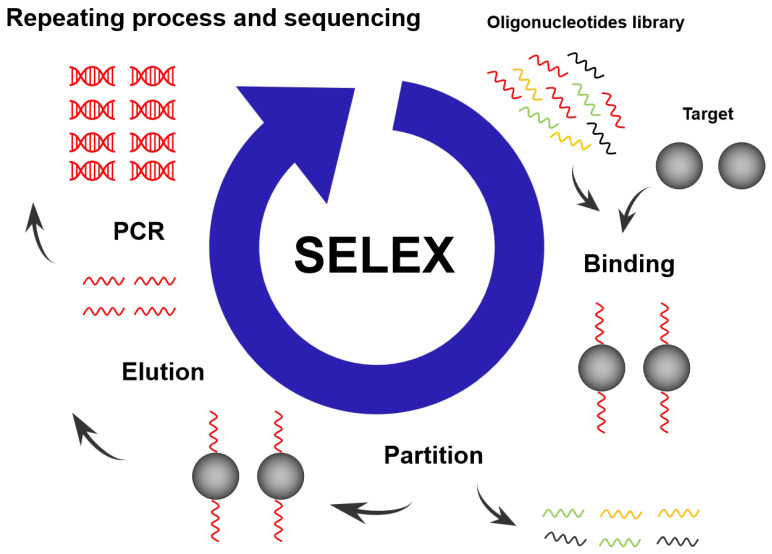
Schematic procedure of the SELEX protocols.

Most aptamers are discovered through a library selection process called Systematic Evolution of Ligands by Exponential Enrichment (SELEX) [1,2]. The working principle of the SELEX is to chemically synthesize a library of single-stranded oligonucleotides as a starting library to incubate with the desired target molecule in vitro to screen for the best aptamer–target binding [27]. The binding of the target with the nucleic acid sequences in the mixture eliminates the nucleic acid sequences that are not bound to the target. Nucleic acid sequences that bind to the target are isolated and amplified using a polymerase chain reaction (PCR) [28,29]. The PCR amplification creates a new pool of nucleic acid sequences, which are then subjected to a subsequent round of the selection process. The initial rounds of screening usually take a longer time, and their screening conditions are more lenient compared to later ones [30]. After the first few rounds of screening, the buffer conditions are usually altered to control the volume of the target–aptamer complex and the binding time of the target to the aptamer to achieve the conditions for creating an aptamer with a high affinity for the target. In addition, the presence of non-specific nucleic acid sequences in the mixture favours the selection of nucleic acid aptamers with a high affinity. At the same time, for some target molecules, the presence of monovalent or divalent cations in buffer solutions can greatly reduce non-specific binding [31]. It is also important to remove non-specific binding sequences from the library using pre-negative selection by incubating the library with the selection matrix, only in the absence of target molecules. After repeated screening and amplification, some nucleic acids that do not bind or have a low affinity for the target reactants are eliminated, and the remaining nucleic acids with a high affinity for the target are isolated. After several rounds of SELEX processes, the purity of nucleic acids with a high affinity for the target is enriched until they occupy the majority of the nucleic acid pool (>90%). As shown in Figure 2, after 5–20 rounds of SELEX selection processes, the selected nucleic acid aptamers with a high affinity to the target were cloned into appropriate vectors and sequenced.

Four factors are critical in selecting nucleic acid sequences that bind to target molecules: the type of randomization, the length of the random sequence region, the chemistry of the nucleic acid library, and the utility of the constant region [32]. During the selection process, the nucleic acid library is processed with the target in the appropriate buffer and at a suitable temperature for the desired application. In vitro SELEX procedures have been widely used to study the nature, function and structure of nucleic acid aptamers. This technology plays an important role in studying molecular recognition and molecular evolution [33]. In order to quickly find residues in the target molecule that cannot be changed without altering the function of the target, in vitro SELEX technologies using DNA and RNA ligands have been proven to be a good approach [34]. One of the most important steps in SELEX technology is the strategy of separating the nucleic acid sequences that are not bound to the target from the sequence library. Therefore, the advancement of SELEX technologies predominantly relies on the improvement of the efficiency and accuracy of separation (or partitioning) of target-bound and non-binding nucleic acid sequences from the oligonucleotide library.

### 2.3. Separation of Target-Bonded and Non-Binding Aptamers

Main separation techniques for conventional aptamer screening technologies are capillary electrophoresis (CE) SELEX, Cell-SELEX, nitrocellulose membrane filtration SELEX, microbead/magnetic bead SELEX, microfluidic microarray SELEX, microcolumn SELEX, and so on. In this part, we will focus on CE-SELEX, Microfluidic SELEX, and Cell-SELEX technologies.

#### 2.3.1. Capillary Electrophoresis SELEX Technology

Capillary electrophoresis SELEX technology is one of the most important methods applied to screen high-affinity nucleic acid aptamers [35]. The principle of CE-SELEX technology is to incubate a nucleic acid library with a target protein and then pass it through a high-voltage electric field within a capillary, causing the unbound nucleic acid sequence to separate from the bound nucleic acid–target protein complex due to the difference in migration rates. Bound nucleic acid–target protein complexes were collected as the end of the capillary, subsequently amplified by PCR to enrich the bound aptamers for the subsequent screening round. Usually the CE-SELEX requires one to four rounds of screening to obtain high-affinity nucleic acid aptamers, which significantly shortens the selection process from weeks to days and are much more effective than the routine SELEX that normally requires several months for 8–15 or more rounds [36]. In CE-SELEX technology, the environment for target proteins and nucleic acid libraries is more liberalized, thus creating a free environment for target proteins and nucleic acid aptamers. This condition makes the probability of non-specific binding of target proteins to nucleic acids lower and not affected by spatial site-blocking during their interaction. CE-SELEX technology was subsequently improved, resulting in the non-equilibrium capillary electrophoresis of equilibrated mixtures (NECEEM) SELEX technology, equilibrium capillary electrophoresis of equilibrated mixtures (ECEEM) SELEX technology, and non-SELEX technology [37,38]. Recently, one-round pressure controllable selection (OPCS), based on conventional CE-SELEX technology, has also been developed [39]. OPCS-SELEX technology enables the simultaneous incubation of nucleic acid libraries with two target proteins, which can provide competitive pressure on each other. Subsequently the complexes formed by these two proteins and the nucleic acid library were separated and collected by high-resolution CE. The enhancement of the efficiency and effectiveness of the OPCS-SELEX technology is achieved by adjusting the concentration ratio of target proteins to improve the affinity and specificity of the nucleic acid aptamer. In addition, the improvement can also be achieved by introducing high concentrations of predatory proteins. The great advantage of OPCS-SELEX technology is that it simultaneously obtains nucleic acid aptamers for two proteins with high affinity and high specificity in a single round, while CE-SELEX technology requires three rounds under the same conditions to obtain nucleic acid aptamers for each protein target [40].

#### 2.3.2. Microfluidic SELEX Technology

Microfluidic technology enables the precise manipulation and control of small fluid volumes within microfabricated devices, making it possible to develop highly efficient and sensitive analytical screening platforms. Integrating microfluidics with aptamer selection technology, Microfluidic SELEX, can precisely control fluids and temperature and complete mixing, incubation, and partitioning from the starting aptamer library to the subsequent round on a single microdevice. It offers advantages over conventional SELEX like a reduced sample volume, improved target-binding capacity, enhanced selection stringency, and automated processing for improved throughput. These advantages have led to various microfluidic-based SELEX techniques that further improve the efficiency and speed of aptamer screening. Further information can be found in latest review articles [29,41,42]. Despite these, some great challenges remain for Microfluidic SELEX, such as the technical complexity of integration of microfluidic platforms and its scaleup and standardization for real-world applications.

#### 2.3.3. Cell-SELEX Technology

Cell surfaces have proteins or glycans which allow nucleic acid aptamers to be screened with purified proteins to recognize target proteins in living cells. Cell-SELEX technology typically uses cells with a characteristic protein expression as target cells, while cells with no or less expression are used for negative selection [43,44,45,46,47]. Cell-SELEX is easy to perform in most biological labs and does not require specialized tools. Cells can be easily partitioned by centrifugation, leaving unbound aptamers in suspension. The cell-bound aptamers are eluted by incubating at high temperatures then separating by centrifugation. Aside from its simple protocol, Cell-SELEX is advantageous because of its authentic target presence on the cell surface and variety of targets (e.g., cancel cells, T cells, bacteria, and viruses). However, the nucleic acid aptamers screened by Cell-SELEX usually do not meet the requirements due to the low specificity of the nucleic acid aptamers. The reason for this is that such cells used for positive and negative screening usually differ in addition to the target protein. To address this, great efforts have been made to improve Cell-SELEX technology. Among them, an approach method proposed by Pleiko et al. [48] is particularly remarkable. They utilized the functional genomics FASTAptamer toolbox and the bioinformatics tool edgeR to investigate binding variability between nucleic acid libraries and positive and negative screening cells. Based on the informative metrics about the selection process achieved with the toolbox FASTAptamer and the tool edgeR, all the sequences in the final nucleic acid library interact with the live cells and were selected for Cell-SELEX to realize a fast and high-throughput aptamer screening protocol against live cells.

## 3. Aptasensors

### 3.1. Aptasensors

**Figure 3:**
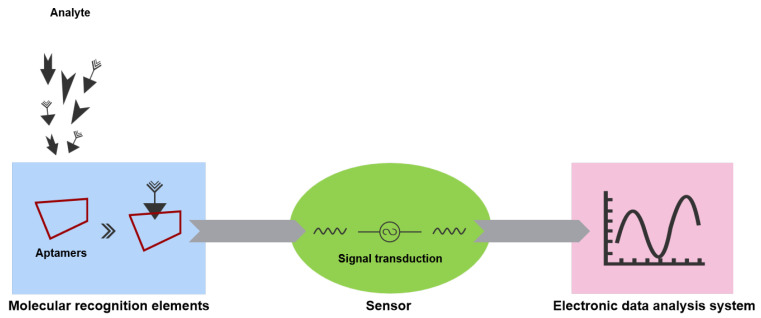
Schematic diagram of the working principle of the aptasensor.

In general, a conventional biosensing device can be broadly defined as a device that converts physical, chemical, or biological events into measurable signals. Biosensors consist of three main components: the biomolecular recognition element, the sensor, and the signal processing and data analysis system. A biomolecular recognition (sensing) element, usually borne by aptamers, antibodies, enzymes, proteins, cellular receptors, tissues, microorganisms, etc., is a biologically derived material or biomimetic element that provides selectivity for a target molecule or a target substance. Sensors can convert molecular recognition events into measurable signals through different transduction mechanisms. After receiving these measurable signals, electronic data analysis systems process the signals accordingly and visualize the data obtained from these processes [49]. Aptasensors act as molecular recognition elements through aptamers because aptamers selectively bind to target molecules [50,51,52]. As shown in Figure 3, when a target molecule in a sample binds to an aptamer, this binding process is converted into a detectable signal through various mechanisms, which is then quantified by the sensor element. Subsequently, the presence of the target molecule in the sample or its specific concentration can be determined [53].

In aptasensors, molecular recognition is a key step in their functioning. The recognition elements for the aptasensors at the early stages were obtained by natural isolation from the biological system. With advances in SELEX technology, molecular recognition elements for aptasensors can be synthesized in the laboratory [54]. The use of aptamers as probes in biosensing devices makes aptasensors uniquely suited for applications, such as blood testing and pathogen detection [55].

Aptasensors have better properties than traditional biosensors that use antibodies as recognition elements [56]. Aptasensors have a higher affinity and specificity for target molecules and target substances. Aptamers can be chemically modified with conformational changes in the target molecule [57]. In addition, some aptamers can be used to obtain specific target molecules by adding specific substances to change their properties. The unique advantage that aptamers can undergo multiple changes in properties allows aptamers to be used in a wider range of applications without compromising their high affinity at the same time [58,59,60,61,62,63]. Aptamers can easily be chemically modified and labelled, enabling aptasensors to be widely used in clinical medicine and medical diagnostics and mass produced and reused [64]. Aptasensors are extremely stable compared with antibodies and can be used multiple times when specific substances are added to regenerate the functionality of immobilized biological components [65]. In addition, aptasensors can be developed for a wide range of target molecules or target substances, and multiple transduction mechanisms can be applied. By applying different transduction mechanisms, aptasensors can be classified into optoelectronics [66,67,68,69,70,71,72], field-effect transistors (FET) [73,74,75,76,77,78,79,80,81,82,83], electrochemical [84,85,86,87,88,89,90,91,92,93,94] aptasensors, and other types of sensors. Aptasensors based on optical, field-effect transistor, and electrochemical transduction mechanisms will be discussed in the following section.

### 3.2. Optical Aptasensors

Optical aptasensors are new types of biosensors based on the use of nucleic acid aptamers as recognition elements, combined with the development of optical detection technology. Optical aptasensors work by capturing signals generated by the interaction of a biometric element with a target molecule and converting the signals into optical signals that are subsequently detected and analyzed [95]. Optical aptasensors use an optoelectronic transduction which allows for rapid and highly sensitive detection and have been widely used in the fields of clinical diagnosis, environmental monitoring, and food safety [96,97,98,99]. Below, the focus will be on fluorescent aptasensors, colorimetric aptasensors, and surface plasmon resonance (SPR) aptasensors.

#### 3.2.1. Fluorescent Aptasensors

**Figure 4:**
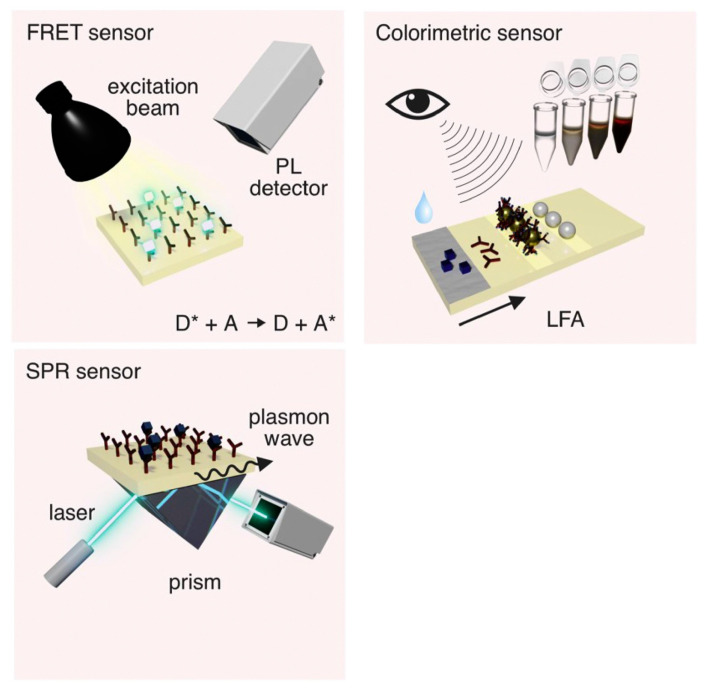
Illustrations of optical biosensing methods utilizing aptamers as recognition elements. Reprinted with permission from Ref. [108]. Copyright 2020, Elsevier.

Fluorescence-based optical aptasensors are constructed in such a way that when a nucleic acid aptamer binds specifically to a target molecule or a target substance, the aptamer–target binding causes a change in fluorescence intensity of the fluorescent material, which is used for monitoring the interactions between the aptamer and the target analytes. Fluorescence-based detection is easy to implement among other optical detection techniques. One of the common fluorescence techniques is the Forster resonance energy transfer (FRET). As illustrated in Figure 4, the principle of FRET involves a radiation-less energy transfer process in which an energy-excited fluorophore (donor) transfers energy to another molecule (acceptor) over a long distance, typically 1nm and up to 10nm via a dipole–dipole interaction [100]. FRET is particularly effective in the detection of interacting membrane proteins and has been used to monitor dynamic processes of protein–protein interactions in living organisms, which is not possible with other conventional monitoring methods. FRET has also been used with monoclonal antibodies, which has led to the better understanding of protein structures in solution, biofilms, and cell surface mapping on immune cells [101]. Moreover, FRET has been widely exploited in DNA sequencing and polymerase chain reactions [102], together with a variety of novel materials for sensing. Among the most widely used materials in fluorescent aptasensors, graphene oxide (GO) has a strong light quenching ability, good dispersion, and biocompatibility [103,104]. GO-based aptasensors have demonstrated great sensitivity and selectivity for DNA analysis by effectively quenching the fluorescence of quantum dots (QDs) when interacting with single-stranded DNA [105,106]. Because of their simple operation, high sensitivity, and non-destruction of the target substance, fluorescent aptasensors have been widely used in a variety of detection and analysis of DNAs and proteins, such as the quantitative analysis of protein biomarkers [107]. Some very recent developments in fluorescent aptasensors have been summarized in Table 2, including target biomarkers, working principles, aptamer immobilization approaches, and the limit of detections, as well as the device features or potential applications.

#### 3.2.2. Colorimetric Aptasensors

Colorimetric aptasensors are a type of aptasensor based on colorimetric bioassay, and they have received more attention in recent years. The principle of a colorimetric bioassay is the detection of the target substances by a colour change using the naked eye and simple instruments (as shown in Figure 4). Colorimetric aptasensors have demonstrated rapid diagnostic capabilities without complicated instrumentation [109]. Colorimetric bioassays are widely used in point-of-care diagnostics and field testing because of their simplicity, low-cost, and effectiveness [110,111]. With the advances of microfabrication and electronic technologies, applications of colorimetric aptasensors have been dramatically expanded with the advent of smart electronic devices, and colour detection of aptasensors have been successfully integrated with commonly used devices such as smartphones, digital cameras, and flatbed scanners [112,113,114]. However, colorimetric aptasensors still face some challenges. For example, colorimetric aptasensors are not as sensitive and accurate as other detection methods in the detection of cancer cells in blood samples [115,116]. In addition, colorimetric aptasensors are highly influenced by the background illumination of the sample matrix and take a long time to be fabricated [117,118]. In clinical diagnostics, colorimetric aptasensors are currently difficult to perform simultaneous multiple target assays with [119].

**Figure 5:**
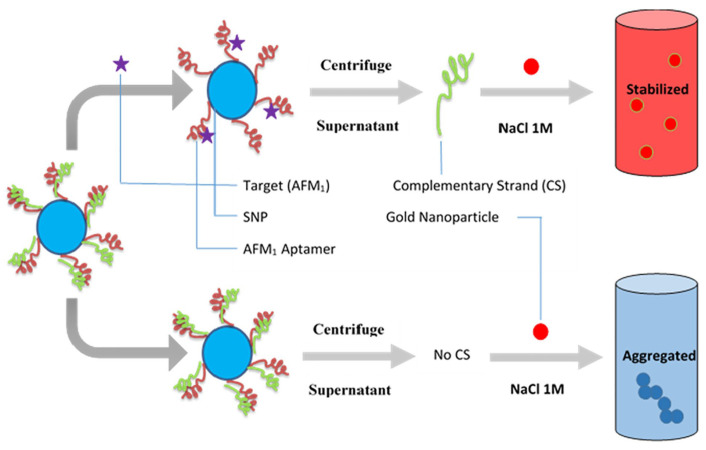
Illustration of the Au nanoparticles (AuNPs)-based colorimetric aptasensor for detection of AFM1. Biotin-labelled AFM1 aptamer and its complementary strand (CS) immobilized onto the surface of silica nanoparticles (SNPs) through streptavidin–biotin (SA-Bio) interaction. Preservation of AuNPs against NaCl-induced aggregation by detaching complementary strand (CS) from dsDNA-modified silica nanoparticles (SNPs) following the addition of the target AFM_1_, and the colour of the sample remains red (upper). Salt-induced aggregation of AuNPs occurs in the lack of the target AFM_1_, and the colour of sample becomes purple as dsDNA-modified SNPs are stable and CS cannot bind to AuNPs. Aptamers work as recognition probe and AuNPs as colorimetric indicators. Reprinted with permission from Ref. [120]. Copyright 2021, Elsevier.

**Figure 6:**
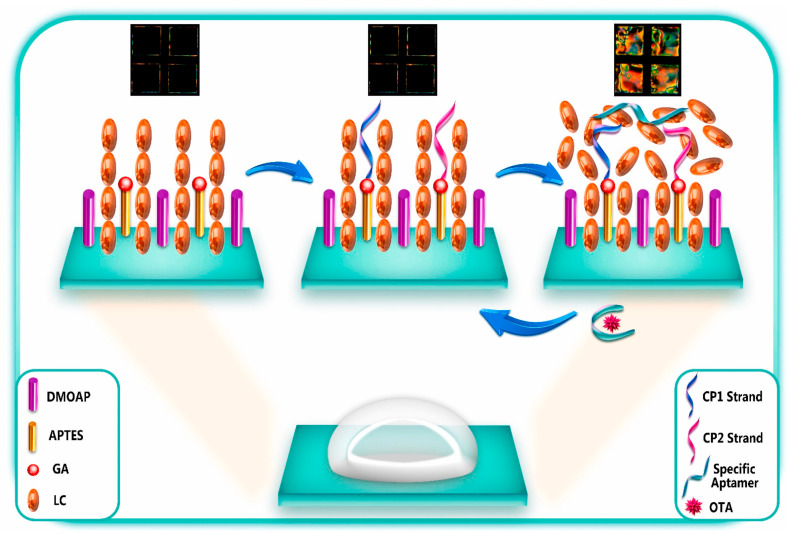
Schematic representation of the LC-based aptasensor to sensitively monitor OTA. The π-shaped DNA structure on the platform substrate disturbed the homeotropic orientation of LC molecules, inducing a bright texture image. In the presence of OTA, the specific aptamer interacted with the target that achieved the vertical alignment of LCs, obtaining a dark texture image. Reprinted with permission from Ref. [121]. Copyright 2021, Elsevier.

As demonstrated in Table 2, among very recent advances in colorimetric aptasensors, gold nanoparticles (AuNPs) and liquid crystal (LC)-based aptasensing strategies have been extensively investigated with significant achievements. The sensing strategies for AuNPs and LC-based aptasensors are further illustrated in Figure 5 and Figure 6.

#### 3.2.3. Surface Plasmon Resonance Aptasensors

As illustrated in Figure 4, the surface plasmon resonance (SPR) aptasensors measure any change in a refractive index proportional to the amount of the target analyte over the process of its binding with the aptamer. The principle of SPR technology is to monitor the change in resonance angle (or refractive index) at the metal surface caused by the specific binding between the substance (e.g., aptamers) immobilized on the metal surface and the target molecule in a sample for biological characterization [122]. Evanescent waves are formed when light waves travelling in a medium undergo a total internal reflection at the interface of the metal and medium, and free electrons (so-called surface plasmons) are generated on the surface of the metal. When the frequency and wave number of two waves are equal, the two resonate, the incident light is absorbed, the energy of the reflected light decreases sharply, and a resonance peak will appear on the reflection spectrum. When the number of target molecules adsorbed on the metal surface or the configuration of the target molecule changes, the change in the peaks on the reflectance spectra can be used for the detection and real-time monitoring of intermolecular interactions without labelling [123,124,125,126]. SPR aptasensors are now widely used in biological research, environmental monitoring, food safety, and clinical diagnosis [127,128,129,130,131]. However, SPR aptasensors also have the disadvantage of being more expensive to manufacture, as a layer of metallic gold usually needs to be deposited to cover the surface of the device [132]. SPR aptasensors are also highly affected by temperature changes.

**Figure 7:**
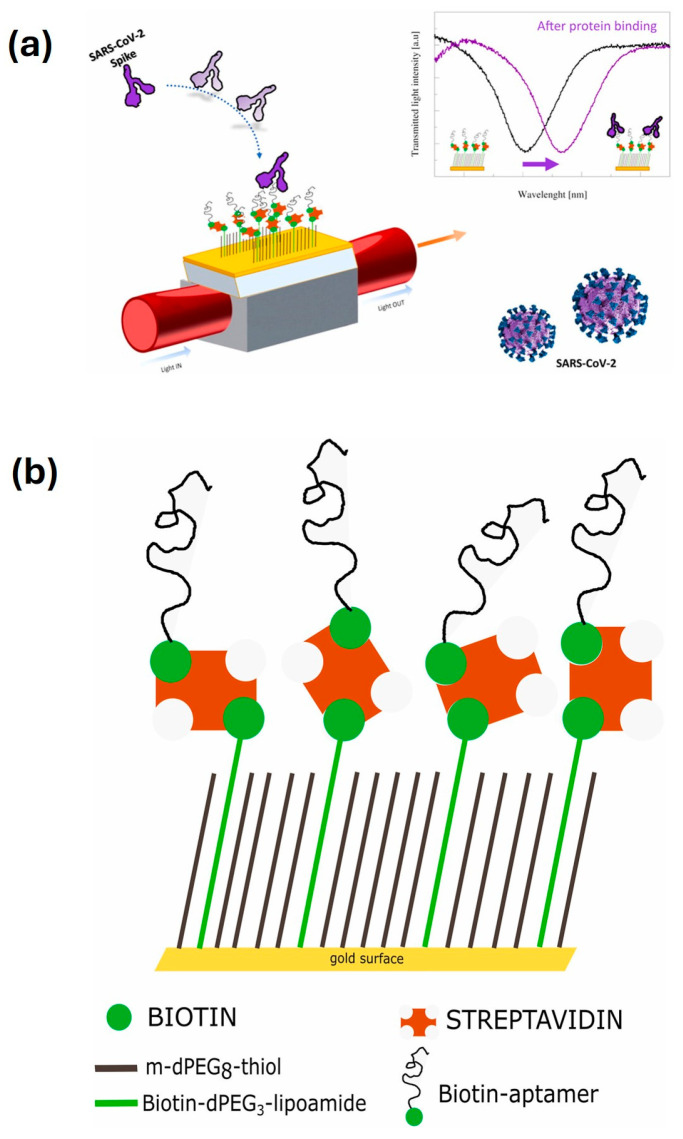
(Left) SARS-CoV-2 spike protein detection through a plasmonic D-shaped plastic optical fibre aptasensor. (**a**) Illustration of working principle of the SPR sensing based on red shift in resonance wavelength upon the binding between the aptamer and SARS-CoV-2 spike protein. (**b**) Illustration of immobilization of the aptamer. Reprinted with permission from Ref. [133]. Copyright 2021, Elsevier.

**Figure 8:**
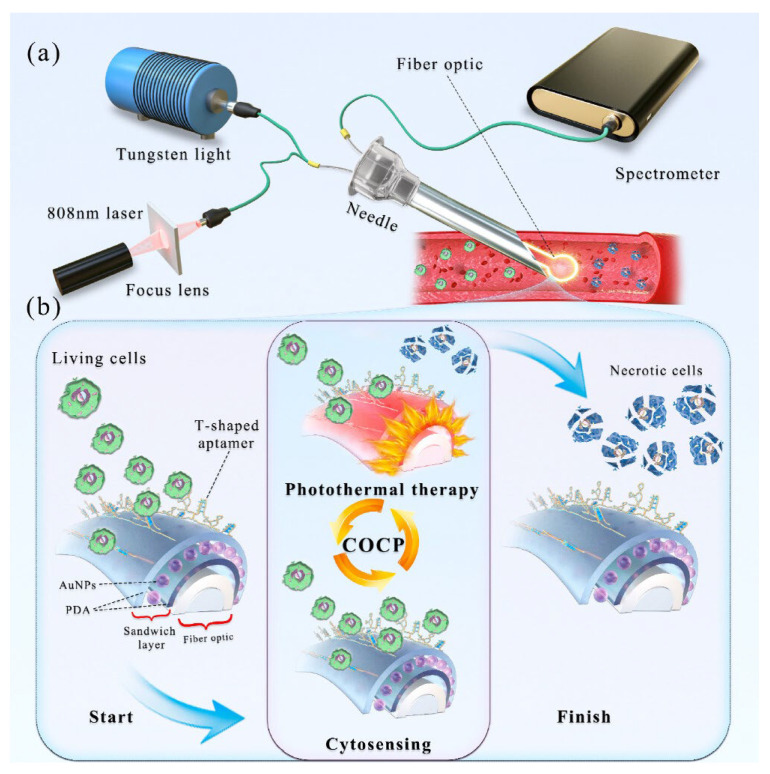
(Right) Illustration of the concept of a circuit of cytosensor–photothermal therapy (COCP) through a fibre optic (FO) LSPR-based aptasensor coating with a sandwich layer. (**a**) The set up of the FO-LSPR. (**b**) Working principle of the COCP for cancer cell capture, kill, and release. The sandwich layer was polydopamine/gold nanoparticles/polydopamine (PDA/AuNPs/PDA). Reprinted with permission from Ref. [134]. Copyright 2024, American Chemical Society.

Despite the challenges in manufacturing SPR aptasensors with metallic gold coatings, gold nanoparticles, nanofilms, and even nano-seed-based sensing strategies still prevail over others for SPR apasensors. It is also noticeable that different-shaped optical fibre SPR probes have been investigated to enhance the SPR aptasensor performance, as shown in Table 2. Figure 7 demonstrated the working principle of SARS-CoV-2 spike protein detection through a plasmonic D-shaped plastic optical fibre aptasensor [133]. What is more, Kong et al. [134] designed a localized surface plasmonic resonance (LSPR)-based aptasensor for a circuit of cytosensing-photothermal therapy (COCP), which can capture and kill the cancer cells and then release dead cells into blood circulation. The COCP was achieved by coating a novel sandwich layer of polydopamine/gold nanoparticles/polydopamine (PDA/AuNPs/PDA) around the Ω-shaped fibre optic (Ω-FO), as shown in Figure 8.

**Table 2:** Summary of recent advances in optical aptasensors.

Analytes	Working Principles	Aptamer Immobilization	Detection Method	Sensitivity (LoD)	Features/Potential Applications	References
Tau441 protein	Changes in the number of target molecules bound to the aptamer-modified biosensor tip cause a shift in the interference pattern that can be measured in real time	Biotin-aptamer was immobilized onto streptavidin (SA) biosensor tips	Biolayer interferometry method	6.7 nM in vitro	Highly selective, portable, label-free, and real-time monitoring	[135]
*E. coli* O157:H7	The specific binding between immobilized **peptide** aptamers on the cavity surface and bacteria’s surface structures (e.g., LPS) induces a shift in the minimum in the optical transmission spectrum	Optical fibre surface was functionalized with extended salinization using APTES and with peptide aptamers immobilized on its surface	Microcavity in-line with Mach–Zehnder interferometer (μIMZI)	10 CFU/mL for *E. coli* O157:H7	Highly specific and sensitive, label-free, and lowest analyte volume	[136]
Proteus mirabilis (pathogen)	The amino-modified aptamers against P. mirabilis were conjugated to the surfaces of SiC quantum dots (QDs) for bacteria recognition; the aptamer with an affinity for target protein can bound to P. mirabilis, which causes a decrease in the fluorescence intensity of DNA-SiC QDs (fluorescence quenching strategy)	The carboxyl groups on the surface of SiC QDs and amino-modified DNA aptamers had a condensation reaction by EDC/NHS coupling to form fluorescence aptasensor	Fluorescent detection	526 CFU/mL with a linear range of 103 to 108 CFU/mL	Potential tool for identification of P. mirabilis in forensic food poisoning cases	[55]
Digoxin (DGX)	The PVA hydrogel acted as a fluorescent probe. The fluorescence intensity of the hydrogel was quenched by aptamer-stabilized **Au nanoparticles (NPs)** as energy acceptors; upon addition of digoxin, aptamer/drug complex was formed, and the fluorescence of the hydrogel was restored because of destabilization and aggregation of AuNPs in the presence of salt	Aptamer of DGX is physically adsorbed on the surface of the 20nm AuNPs and then mixed with solutions of NaCl and PVA with or without DGX for detection	Fluorescent detection with on/off/on strategy	2.9 ng/L with a linear range of 10–1000 ng/L	High selectivity, eco-friendly, and rapid operation	[137]
Zearalenone (ZEN, one of the most prevalent mycotoxins)	The 17-nt fluorophore Cy5.5-labelled complementary DNA strand and ZEN competitively bind with the aptamer immobilized on the fibre, enabling **signal-off** fluorescent detection	A 40-nt ZEN-specific aptamer (8Z31) is covalently immobilized on the optical fibre by coupling its amino group at the 5′ end with aldehyde group introduced on the fibre surface	Fluorescent detection	18.4 pM (equivalent to 29.3 ppt) with a semi-log linear range of 10 pM–10 nM	Extremely high specificity, regenerated 28 times, decreased cost	[138]
Kanamycin (Kana, an important antibiotic used in veterinary medicine)	The quencher TTQF binds strongly with ssDNA, whereas it exhibits very weak interactions with dsDNA; by subtly designing the structure of the DNA aptamer probes, the target-induced fluorescence signal is amplified to different levels for sensing	Fluorescein labelled at the end of the aptamer as FRET donor (an organic quencher TTQF as acceptor)	Fluorescence spectra (FRET)	10 nM with a linear range of 10 nM to 3 μM	High specificity, selectivity, and stability for antibiotic determination in food and environment	[139]
Endotoxin	Upon binding with endotoxin, the aptamer undergoes conformation changes and decreases the distance between the Au nanoparticle (**AuNP**) and the Au nanofilm (**AuNF**), resulting in the red shifting of scattered light spectrum	The aptamer has an amine group at the 5′ end coupling to the SAM layer on the AuNF and a thiol group at the 3′ end, which forms a covalent bond with AuNP	Time-averaged **scattering** spectra OR colour camera for single-particle mode	15–31 EU/mL (ensemble mode) OR single-particle resolution	Reusable, highly selective	[140]
Lipopolysaccharide (LPS) biomarker of Gram-negative bacteria	Orientation of liquid crystal (**LC**) molecules at the LC-aqueous interface can be changed upon binding of aptamer with LPS in the mixture solution, inducing optical appearance from bright to dark	LC molecules are immobilized and formed onto glass slides for optical sensing cells	Polarized light microscopy	Ultralow, 0.4 pg/mL for LPS biomarker, and 27 cfu/mL for E. coli bacteria	Simple, label-free, high sensitivity and specificity, and rapid and reliable detection of Gram-negative bacteria	[141]
Ochratoxin A (OTA, the most dangerous mycotoxin)	The aptasensing strategy was based on the conformational switch of the immobilized π-shaped DNA aptamer structure on the glass substrate in presence of the target; a shift in the orientation of **LCs** from random to homeotropic state led to the change in optical appearance of the sensor platform from bright to dark	Two complementary strands of aptamers (CPs) were first immobilized onto glass substrates, subsequently modified with DMOAP/APTES/GA, and then incubated with solution of OTA-specific aptamer for immobilization	Polarized light microscopy	Detecting OTA at the ultra-trace level as low as 0.63 aM	Portable and real-time sensing probes with high performance for food safety control and clinical application	[121]
Prostate-specific antigen (PSA, prostate cancer biomarker)	The principle of PSA detection by the aptasensor is based on the changes in the directional regularity of **LCs** caused by the specific aptamer at the CTAB-arranged LC-aqueous interface; the optical vision of the aptasensor background changes from colourful to dark by forming the aptamer–PSA complex that induces the re-arrangement of **LC molecules** from disordered to homeotropic direction	The cleaned glass slide was modified by soaking in DMOAP solution, CTAB + 5CB mixture solution, and then aptamer solution, subsequently; afterward, the solution of PSA was pipetted on to the aptasensor for detection	Polarized light microscopy	0.148 pg/mL with a range of 1–1200 pg/mL	Label-free, simple and low-cost, highly sensitive aptasensor for diagnosis of PSA	[142]
Insulin	Insulin-binding DNA aptamer (IGA3) is a guanine (G)-rich DNA that forms an anti-parallel G-quadruplex and binds insulin selectively. Nickel (II) salphen complex modified with piperidine side chain [NI(II)-SP] acted as the ligand to the immobilized aptamer via non-covalent π-π stacking between aromatic rings of folded single-strand IGA3 aptamer and square planar salphen ligand; a colour change from yellow to brownish orange is induced upon the binding of insulin with the immobilized IGA3-Ni(II)SP complex	Insulin-binding DNA aptamer (IGA3) was employed as the synthetic receptor and conjugated to the amine-modified porous silica microparticles (PSiMPs) via imine covalent bonds using glutaraldehyde crosslinker	Fibre optic reflectance spectra	3.71 μIU/mL with a linear range of 10–50 μIU/mL	High selectivity, rapid response, promising for insulin monitoring simply by observing the aptasensor colour change.	[143]
Aflatoxin M_1_ (AFM_1_)	Preservation of **AuNPs** against NaCl-induced aggregation by detaching complementary strand (CS) from dsDNA-modified silica nanoparticles (SNPs) following the addition of target, and the colour of sample remains red; salt-induced aggregation of AuNPs occurs in the lack of AFM_1_, and the colour of sample becomes purple as dsDNA-modified SNPs are stable and CS cannot bind to AuNPs (as demonstrated in Figure 5)	Biotin-labelled AFM_1_ aptamer and its complementary strand (CS) immobilized onto the surface of silica nanoparticles (SNPs) through streptavidin–biotin (SA-Bio) interaction. Aptamers as recognition probe and AuNPs as colorimetric indicators	Colorimetric detection	30 ng/L with a linear range of 300–75,000 ng/L	Simplicity (detected with the naked eye), better selectivity by using CS of aptamer	[120]
Retinol-binding protein 4 (RBP4) (a biomarker for the early diagnosis of T2DM)	Retinol-binding protein aptamer (RBP-A) physically adsorbs on the AuNPs surface and stabilizes the **AuNPs** against NaCl-induced aggregation; upon the addition of RBP4, RBP-A binds to RBP4 and detaches from the AuNPs surface, leaving the AuNPs unprotected. Addition of NaCl causes aggregation of AuNPs, leading to a colour change in the AuNPs solution from red to purple/blue	RBP-A is physically adsorbed on the surface of the AuNPs through van der Waals and hydrophobic interactions	Colorimetric detection	90.76 nM	Simple yet effective, specific, and rapid detection of RBP4	[144]
Interleukin-6 (IL-6) and C-reactive protein (CRP) (sepsis biomarkers)	Aptamer-functionalized **AuNPs** used for detection of CRP and IL-6 biomarkers; using colorimetric assay, aggregation value of AuNPs in presence of the target was determined in two phases: in solution and on μPADs; and a competitive reaction between target and AuNPs was performed to bind to the aptamer, bare nanoparticles were aggregated in the presence of salt, and the concentration of the analyte was measured according to the amount of colour change due to aggregation	Aptamer for either CRP or IL-6 protein biomarkers was not modified and physically adsorbed onto the surface of the AuNPs; the aptamer-functionalized AuNPs were mixed with CRP and IL-6 proteins in PBS solution and then incubated for quantification	Colorimetric detection	Linear ranges were 50–1000 mg/L and 1–25 ng/L, and LoD values were 1.9 mg/L and 0.07 ng/L for CRP and IL-6, respectively	Microfluidic paper-based analytical devices (µPAD), simple but highly stable, reproducible, accurate, and recoverable for sepsis diagnosis; determination of two biomarkers with naked eyes and using image analysis	[145]
Mammaglobin proteins at the surface of circulating breast cancer cells (CTCs)	The resonance wavelength and amplitude are impacted and shift proportionally to the refractive change induced by the binding between immobilized aptamers and surface proteins of the cells	Thiolated MAMA2 aptamers immobilized on the surface of **Au**-coated optical tilted fibre Bragg gratings	**SPR** with tilted fibre Bragg gratings (TFBGs) sensitivity enhancement	49 cells/mL (down to 10 cells/mL when AuNPs used for signal amplification)	Highly sensitive, selective, and quick response for early-stage metastatic cancer diagnosis	[146,147]
*Plasmodium falciparum* glutamate dehydrogenase (PfGDH, malaria biomarker)	**U-bent** optical fibres as a LSPR probe creates higher-order modes, resulting in a higher penetration depth of the evanescent field; the probe measures the refractive index changes, initiated by the binding of PfGDH to the aptamer	The **U-bent region** of plastic optic fibres (POF) was de-cladded and then sputtered with gold for LSPR; the thiolated-PfGDH aptamer NG3 and 6-mercapto 1-hexanol (MCH) were then immobilized on the **Au** surface via incubation	LSPR detecting refractive index changes	264 pM with only 175 mL of the sample used	Low-cost, simplistic, robust aptamer-based stable recognition system, and multiplexing capability	[148]
SARS-CoV-2 spike protein	A specific aptameric sequence was immobilized on short polyethyleneglycol (PEG) interface on gold nanofilm deposited on a **D-shaped** plastic optical fibre (POF) probe, and the binding of SARS-CoV-2 spike glycoprotein with the aptamers induced a clear red shift in the resonance wavelength, which is a very sensitive surface plasmon resonance (SPR) phenomenon being monitored for spike protein detection, as demonstrated in Figure 7a.	A mixed layer obtained from a mixture of PEGthiol and BiotinPEGlipo was prepared on Au nanofilm, and a streptavidin coating was next performed; finally, a biotin-modified aptamer was immobilized on the streptavidin layer, as demonstrated in Figure 7b.	SPR	37 nM, with a range of 25–1000 nM	High specificity, rapid response, low-cost, and compact, portable diagnostic tool	[133]
Cancer cells	In the processes of cytosensing, cancer cells were selectively captured onto the T-shaped aptamer-coated **Ω-shaped** fibre optic (Ω-FO) surface; in the following photothermal therapy (PTT) process, high local temperature induced necrosis of cancer cells, which could be released from the FO surface (as demonstrated in Figure 8)	A sandwich layer of PDA/AuNPs/PDA was designed as a coating around the surface of the Ω-FO to enhance LSPR sensitivity and photothermal conversion efficiency; T-shaped aptamers (AS1411 and MUC1) were immobilized on the coating surface to sensitively capture cancer cells in blood circulation system	Localized surface plasmon resonance (**LSPR**) detecting refractive index changes	13 cells/mL	The sandwich layer coating strongly enhanced efficiencies of cytosensor and PTT; significant potential for cancer metastasis inhibition	[134]
Helicobacter pylori (H. pylori)	The optical fibre surface was modified with a layer of AuNPs; then, the polyA-tailed aptamer and the block agent of DNA with secondary structure were fixed on **AuNPs** via the Au-N bond; and when H. pylori is present in the system, the aptamers on the optical fibre surface specifically recognize and capture H. pylori, which caused the intensity change in the LSPR absorption peak due to the refractive index change of the solution.	Original sequence of aptamer was truncated to effectively capture H. pylori; cheaper polyA-tailed aptamers utilized instead of thiolated aptamers; a spacer nucleic acid with short stem-loop structure was adopted to control the aptamer density on AuNPs on the surface of the **J-shaped** optical fibre probe	Localized surface plasmon resonance (LSPR)	45 CFU/mL with a linear range of 1.0 × 10^2^–1.0 × 10^8^ CFU/mL	Easy-to-prepare, high sensitivity, and rapid analysis for detection of pathogenic bacteria in environmental monitoring and disease diagnosis	[149]
Zearalenone (ZEN, one of the most prevalent mycotoxins)	Target analyte-tuned growth of gold nano-seeds (**AuNSs**) coated with unmodified aptamers was derived from the DNA-mediated control of the metal nanoparticle shape and optimized to determine concentration of the analyte according to the LSPR peak wavelength of the grown AuNPs	5 nm AuNSs were modified with both 8Z31 and Z143 aptamers by physical adsorption via incubation together in the mixture solutions	Absorption spectra (LSPR)	∼72 ppb with a range of 10 ppb to 100 ppm	Rapid detection of small molecules based on analyte-tuned growth of AuNS, combined with machine learning-enhanced spectrum analysis	[150]
Four mycotoxins (AFB1, FB1, OTA, and ZEN)	Optical fibres are utilized not only as the carrier to immobilize single-stranded binding protein (SSB) to prepare the sensing region but also as the transducer of the chemiluminescent emission to detect the sensor response; when the fibre sensing probe with SA-Bio-HRP complex on the fibre surface was immersed in the solution containing chemiluminescence substrates, chemiluminescent emission was transmitted through the optical fibre and collected by the photon-counting detector	Firstly, each target and its biotinylated aptamer (Bio-Apt) are mixed in solution for complete specific recognition; then, the fibre sensing probe with immobilized SSB was placed into the mixture solution to make SSB bind to the remaining Bio-Apt molecules that are not recognized by the targets; and afterward, the probe was inserted into a solution to form SA-Bio-HRP complex on the fibre surface	Chemiluminescent emission and photon counting-detector	0.015–0.423 pg/mL with a range from pg/mL to 105 pg/mL for detecting different mycotoxins	Four mycotoxins (AFB1, FB1, OTA, or ZEN) can be simultaneously determined from one run for on-site analysis of real food samples	[151]

### 3.3. Field-Effect Transistor Aptasensors

**Figure 9:**
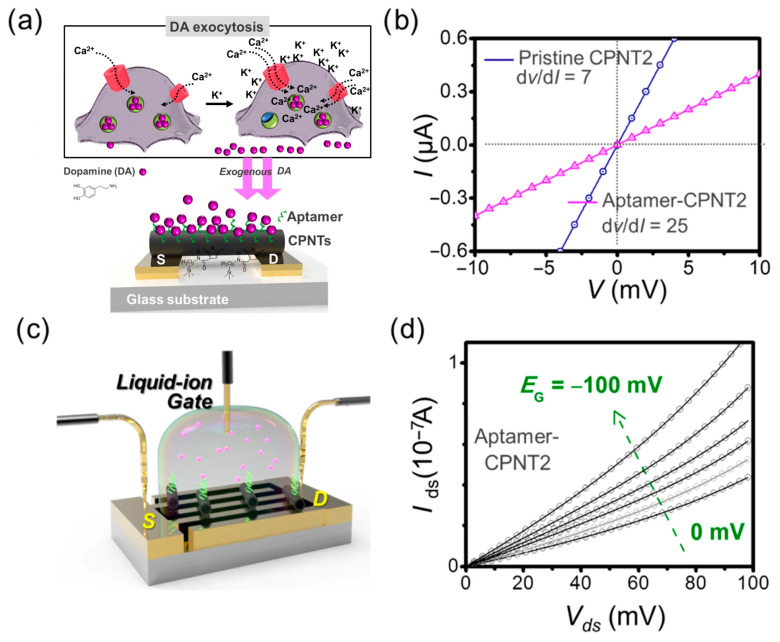
(**a**) Illustration of cell exocytosis for dopamine (DA) release from PC12 cells via rapid Ca^2+^ reflux, accelerated by K^+^ ions (upper) and liquid-ion gated FET aptasensors, using aptamer-conjugated CPNTs for exogeneous DA detection (down). (**b**) Current-voltage (I-V) curves of CPNT2 before and after the introduction of aptamer. (**c**) Schematic illustration of liquid-ion gated aptasensors using CPNTs on the interdigitated microelectrode (IME) array. (**d**) Output characteristics of liquid-ion gated aptasensors with CPNTs. S and D in (**a**) and (**c**) stand for source and drain, respectively. Adapted from Ref. [76]. Copyright 2020, the Springer Nature.

Field-effect transistor aptasensors are sensors constructed based on field-effect transistors with aptamers as the molecular recognition element. The FET is a semiconductor device that uses the electric field effect of the input loop to control the output loop current. The source (S) and drain (D) are connected by semiconductor channels to form a typical FET aptasensor. As illustrated in Figure 9a,c, any adsorption and/or recognition events of a target molecule with the aptamers immobilized on the surface of a channel causes a change in the electric field. Adjusting the gate potential (*E*_G_) at this point can cause the drain current in the FET channel to change (Figure 9b,d). FET aptasensors are used to detect and analyze target molecules by monitoring changes in the drain current [76,152,153,154,155,156,157,158,159,160]. FET aptasensors are inexpensive to manufacture compared to aptasensors with other transduction mechanisms, especially SPR aptasensors, and can be integrated with existing manufacturing processes on a large scale. In addition, as demonstrated in Table 3, FET aptasensors have other advantages, such as the ability to operate at low power, the ever-shrinking design size, and their rapid detection, which makes them promising sensors [161]. Although FET aptasensors have been rapidly optimized over the past few years, there are still significant challenges in improving their detection sensitivity, as this can be affected by various factors [162].

**Figure 10:**
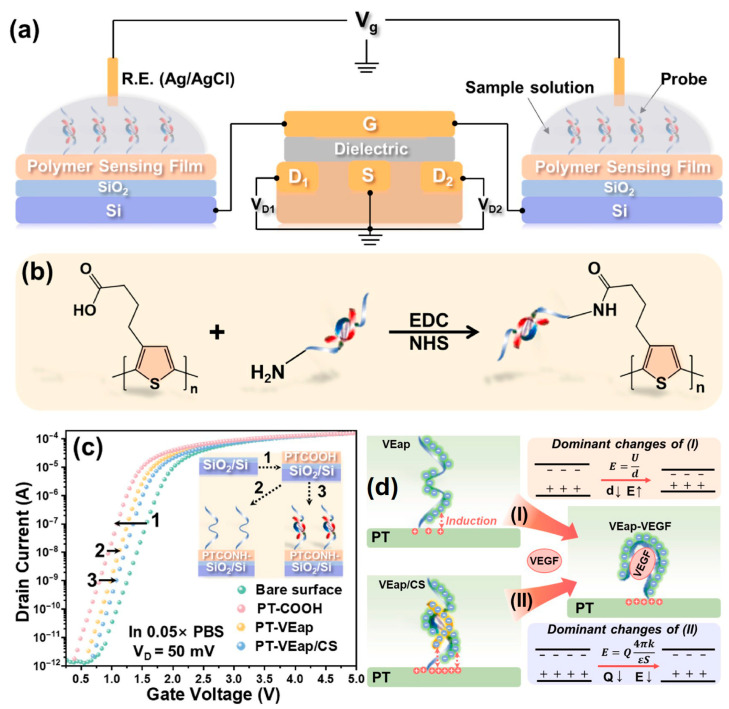
(**a**) Schematic of the remote dual-gated FET sensing system: the two remote gates are connected in parallel to the gates of commercial transistors. (**b**) Functionalization of the PT-COOH film with probe VEap/CS to form PT-VEap sensing surface on the remote gates using EDC/NHS chemistry. (**c**) Representative transfer curves of FETs with remote gates in a 0.05 × PBS buffer solution: bare surface, PT-COOH, PT-VEap, and PT-VEap/CS. The single-strand blue line represents VEap, while the red–blue double-strand chain represents VEap/CS. (**d**) Proposed mechanisms of VEGF responses on PT-VEap (Process I) and PT-VEap/CS (Process II) as remote gates of FET sensors. The primary change in Process I is attributed to the conformational folding of VEap upon VEGF binding, which reduces the separation distance between the plates of the model capacitor. The primary effect in Process II is attributed to the release of the CS along with VEGF binding to VEap, leading to a decrease in the charge stored in the model capacitor. Adapted from Ref. [168]. Copyright 2025, Elsevier.

Due to their phosphate backbone, aptamers are strongly polyanionic in blood plasma or simulated body fluids (SBF). Many proteins have positively charged domains (rich in lysine, arginine, and histidine residues); electrostatic attractions help aptamers approach and orient them toward the protein surface. Mg^2+^, Na^+^, and K^+^ Ca^2+^ ions in blood plasma or simulated body fluids partially neutralize the aptamer’s backbone, which reduces the electrostatic repulsion within the aptamer, helps folding into the stable, 3D-binding structures (G-quadruplexes, hairpins, etc.), and allows for more precise shape complementarity with protein-binding pockets. The polyanionic nature of aptamers also reduces nonspecific binding to negatively charged cell membranes via the repulsion force and improves solubility in aqueous environments. All these features have been extensively exploited for optical aptasensors, as demonstrated by some examples summarized in Table 2. In FET sensors, these features have also been explored to amplify the target-binding signal by rearranging the negatively charged oligonucleotide backbone and associated solution ions near the semiconductor surface, where the net redistribution of the aptamer backbone upon target recognition drives the sensor response, as illustrated in Figure 10 [76,163,164,165,166,167,168,169,170].

**Figure 11:**
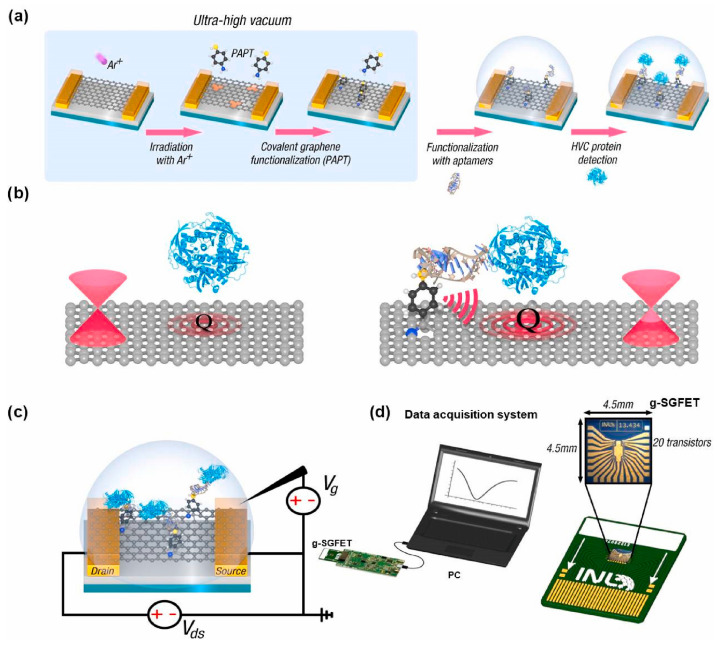
(**a**) Protocol to develop a covalent graphene solution gated field-effect transistor (g-SGFET) aptasensor. The protocol is a two-step process carried out in UHV: i) the creation of single-atom vacancies in the graphene layer of the transistor with an Ar+ ion sputter gun; ii) the evaporation of Langmuir of p-aminothiophenol (pAPT). Then, the platform is functionalized with the selected aptamer specific to the Hepatitis C virus (HCV) core protein. The two final steps are not carried out in UHV but in a liquid environment. (**b**) Molecular antenna effect: the covalently bound pATP linker acts as a molecular antenna enhancing the local polarization of graphene due to the proximity of the aptamer/protein system, setting the position of the Dirac cone. (**c**) g-SGFET functioning scheme: The g-SGFET contains three terminals: the source, drain, and gate electrodes, and the active channel formed by graphene. The charge carriers (electrons or holes) flow through the active channel from the source to the drain. The gate modulates the channel conductivity by applying a gate voltage that moves the graphene Fermi energy up or down in the density of states for positive or negative voltages. (**d**) Real image and dimensions of the g-SGFET wire-bonded to a printed circuit board (PCB) inserted into an electronic platform that can communicate with a computer where the measured data are displayed. Adapted from Ref. [171]. Copyright 2023, Elsevier.

In FET aptasensors, aptamer functionalization methods are essential and can be categorized into physical and chemical approaches. The aptamer physical functionalization approach is based on the physical processes of secondary bonding interactions such as hydrogen bonding, van der Waals forces, electrostatic interactions, π-π stacking, and hydrophobic interactions [165,166,167,168,169,170]. Especially for single-layer-based graphene field-effect transistor (g-FET) aptasensors, molecular crosslinkers, such as 1-pyrenebutyric acid N-hydroxysuccinimide (PBASE) [165,167,169] or p-aminothiophenol (pATP) [171], are often used to enhance the immobilization of aptamers via π-π stacking, as illustrated in Figure 11. The aptamer chemical functionalization methods are based on the chemical processes of forming primary bonds, such as covalent bonding and crosslinking, as demonstrated in Figure 10 and as summarized in Table 3 [170,171,172,173,174,175,176,177,178,179,180,181,182,183,184,185].

In the physical functionalization methods of aptamers, the aptamers physically adsorb onto the surface of the channel and then bind with the target molecules. The operation process is relatively simple, which reduces the possibility of aptamer denaturation and maintains the electrical properties of the channel material. However, in the physical functionalization methods of aptamers, the interaction force between aptamers and channel materials is relatively weak, and it could be difficult to control the distribution and packing orders, which can lead to aptamer dissociation and reduce the detection repeatability and long-term stability of FET aptasensors. When FET aptasensors are chemically functionalized, the channel surfaces normally need to be activated at first with carboxylation, hydroxylation [186], amination, or gold deposition [187]. Subsequently, one end of the linker molecule is covalently bonded to the channel surface, and the other end of the linker molecule is covalently bonded to the aptamer. During chemical functionalization, aptamers are immobilized onto channel surfaces through covalent bonds, which are strong and can improve the detection repeatability and long-term stability of FET aptasensors. However, aptamer chemical functionalization methods require additional sequence design and purification based on specific target molecules and target substances. Furthermore, during the chemical functionalization process of aptamers, the conformational stability of aptamers could be subject to unpredictable interference [188]. Therefore, it is necessary to select the appropriate aptamer functionalization method based on the specific circumstances. Because of their high selectivity, good portability, and low manufacturing cost, FET aptasensors have been well developed and will be further exploited in more fields with better standardization and commercialization [189].

**Table 3:** Summary of recent advances in field-effect transistor (FET) aptasensors.

Analytes	Aptasensor Architecture	Aptamer Immobilization	FET Type	Sensitivity (LoD)	Feature/Special Findings	References
Dopamine (DA, brain hormone)	Sequential conjugation of carboxylated polypyrrole nanotubes (CPNTs) and DA-specific aptamer molecules on the interdigitated microelectrodes (IMEs), and the modified substrate integrated into liquid-ion gating system surrounded by pH7.4 buffer (as demonstrated in Figure 9).	DNA aptamer specific to DA as a gating modulator was chemically attached to the surface of CPNTs.	Liquid-ion gated FET	100 pM	Smaller diameter CPNTs of ca. 120 nm achieved over 100 times higher sensitivity than the larger diameter of ca. 200 nm.	[76]
Dopamine (DA)	Micron-sized electrolyte-gated graphene field-effect transistors (EG-gFETs) were fabricated with silicon wafer via sputtering, lithographic patterning, and ion milling. Then, single-layer graphene (SLG) grown by thermal chemical vapour deposition (CVD) on Cu foils was transferred onto the wafer, patterned with lithography, etched by plasma, and passivated.	EG-gFET arrays were functionalized firstly with 1-dodecanethiol (DDT) for gate passivation and then with crosslinker 1-pyrenebutyric acid N-hydroxysuccinimide (PBASE). Afterward, a 44nt-long DNA aptamer for high affinity to DA with a 5′ amino-link termination was bonded with PBASE on the sensor surface.	Electrolyte-gated graphene field-effect transistors (EG-gFETs)	1 aM with dynamic range of 10^−18^ to 10^−8^ M	Demonstrated highly pertinent for developing novel point-of-care devices that require stable, high-throughput detection of physiologically and clinically relevant dopamine concentrations.	[169]
Hemagglutinin (HA) protein (biomarker for H5N1 avian influenza virus AIV)	The Au electrode of a sensing part as extended gate is functionalized with DNA aptamer specific to HA protein. Surface potential is generated due to a conformational change in DNA aptamer induced by the binding of HA protein. The generated surface potential is transferred with an FET transducer by connecting the sensing part to the gate of a commercial MOS-FET transducer, where the drain current is measured as a function of the gate voltage applied with a reference electrode inserted into the target solution.	The specific DNA aptamer against the HA protein was hybridized with thiol-tagged shorter complimentary DNA strand to enable formation of covalent bonds with the gold electrode using a thiol group. The DNA aptamer was also hybridized with HRP-tagged complimentary DNA strand to create the DNA 3-way junction.	Extended-gate FET	5.9 pM with a dynamic range of 10 pM to 10 nM	First use of an aptamer-functionalized FET for AIV detection.	[80]
Ochratoxin A (OTA, a mycotoxin contaminant)	An array of graphene FETs was integrated on a single silicon chip. A **single-layer graphene** was transferred onto a Si substrate with 300 nm SiO_2_ layer by a wet transfer and then patterned to form graphene channels via oxygen plasma etching. E-beam-assisted evaporation was used to deposit Au electrodes.	Specially designed aptamer for OTA with amino-modified at 5′ end was covalently bound onto the sensor surface via a linker of PBASE molecule. The transverse electrical field was used to increase the density of PBASE linker immobilization on graphene by π-π stacking.	**Graphene** field-effect transistors (gFET)	1.4 pM in a dynamic range 5–500 pM	Real-time, highly responsive, and rapid detection of OTA is achieved. Ionic strength effect on sensitivity to OTA in buffer is demonstrated.	[165]
Ochratoxin A (OTA)	Constructed with high-purity **carbon nanotube (CNT)** films, svelte yttrium oxide dielectric layers, and extensive gold nanoparticles (AuNPs) to form an ultrathin, active channel. Strategic immobilization of DNA aptamers onto **AuNPs** confers the sensor with exceptional selectivity. Interactions between the aptamers and the target molecule prompt electrostatic modifications, leading to a reliable biomolecular response.	Thiolated DNA aptamers specific to OTA were immobilized on the sensor surface through forming Au-S bonds with AuNPs distributed along the CNT network, thereby anchoring it firmly to the FET structure. BSA adsorption was subsequently applied to block any remaining active sites, preventing non-specific binding.	CNT FET	0.2 fM with a range of 8 fM to 80 pM	Label-free, highly sensitive, and rapid detection method for OTA.	[172]
Tetracycline	To construct the FET devices, the source and drain electrodes were prepared by depositing 10 nm Ti and 50 nm Au onto silicon-based **graphene** using a shadow mask and the e-beam evaporation deposition method. The PDMS microfluidic chip was prepared with the aid of photolithography and an inversion process. A Ag/AgCl reference electrode in PBS solution was used as the solution gate through the microfluidic channel.	The ssDNA aptamers were denatured and then injected into the channel and incubated to ensure sufficient fixation of the aptamer, followed by rinsing of the channel with PBS solution and then BSA to remove excessive aptamers and block nonspecific reaction sites.	Camphor−rosin clean transferred **graphene** FET (CRCT-FET)	100 fM with a range of 10 pM to 100 nM.	The carrier mobility of the FET made with ultraclean graphene is improved by more than 10 times compared with the FET prepared by conventional PMMA transfer (CPT) method.	[170]
Tetracycline	The solution-gated **graphene** transistor (SGGT)-based sensor with gate, source, and drain electrode and a monolayer graphene channel connecting the source and drain was prepared following the procedure of substrate cleaning, magnetron sputtering, and wet transfer of graphene. The prepared sensor was put into acetone solution to remove PMMA as the organic carrier for the monolayer graphene. The electrode was then encapsulated with PDMS and silver paste to form a reaction tank.	The aptamer against tetracycline (denoted as APT40) with thiol group at 3′ end form covalent Au–S bonds on the Au gate electrode for immobilization.	Solution-gated **graphene** FET (SGG-FET or SGGT)	2.073 pM	Demonstrated miniaturized aptamer-SGGT biosensor with appropriate detection strategies could provide an ideal portable sensing platform.	[173]
Spike protein of SARS-CoV2	An aluminum contact was deposited on an intrinsic silicon field-effect transistor sensor and then functionalised with APTES, glutaraldehyde (GA), aptamer, and glycine molecules in sequence. The glycine is aimed at terminating any unbound aldehydes which might create non-specific binding sites.	The specific aptamer against spike protein with an amine group attached to the 5′ end was immobilized by reacting with aldehyde of glutaraldehyde, which was already bound with the amine-terminated silane on the sensor surface.	Schottky field-effect transistor	A linear range of 100 fM to 10 pM (75 pg/mL to 7.5 ng/mL)	Feasibility of an aptamer-FET to detect spike protein.	[174]
Hepatitis C virus (HCV)	The **graphene** solution-gated FET (g-SGFET) was fabricated with silicon (Si) wafer (p-type doped with boron) by multiple steps using optical lithography, ion milling, sputtering, and plasma etching, as well as CVD-grown graphene method. Two-step process of graphene functionalization in ultra-high vacuum (UHV) chamber was performed to create single-atom vacancies in transistor graphene layer using ion-sputtering and then to form **covalent bond** with p-aminothiophenol (pATP) linker molecules via evaporation of 27 Langmuir of pATP. The functionalization strategy preserves graphene properties and allows for precise control of the density of immobilized aptamer probe molecules.	A 76 nt-long ssDNA aptamer termed AptD-1312 with a thiohexyl motif at 5′ end was selected as an optimal probe molecule for detection of HCV core protein. A 76 nt-long and thiohexyl-modified ssDNA molecule termed D-ACTG was also used as a negative control. The immobilization of thiol-modified AptD-1312 or D-ACTG onto the p-ATP-functionalized surface of the g-SGFET was performed with denaturation and incubation for crosslinking via disulfide bridge.	**Gra-phene** solution-gated field-effect transistors (g-SGFET or SGGFET)	15.6 aM with a linear range of 10^−14^ to 10^−18^ M in the buffer solution; 90.9 aM in a linear range of 10^−13^ to 10^−18^ M in human blood plasma	Unprecedented sensitivity is due to molecular antenna effect of pATP linker, which can capture a subtle charge transfer at the linker/graphene interface driven by local polarization of graphene by the proximity of the aptamer/protein system. The technology is easily scalable in an inexpensive configuration and transportable to the point of care (PoC) in clinical environments.	[171]
Thrombin (a model biomarker)	Arrays of silicon nanowire field-effect transistors (SiNW FETs) were fabricated from silicon-on-insulator (SOI) substrates.	EHTES organosilane was used to graft thrombin-binding aptamer (TBA) onto the HfO_2_ gate oxide of Si nanowires. The TBA probes molecules with an amine termination at the 5′ end.	Silicon nanowire field-effect transistor (SiNW FET)	Tested at a concentration of 2.7 μM only	Feasible to be co-integrated with CMOS readout circuits in the future.	[175]
Interleukin 6 (IL-6)	The silicon nanowire field-effect transistor (SiNW FET) devices were commercially available. Each n-type SiNW FET device had two nanowires, and each nanowire had a length of 2 μm and a width of 200 nm. After thoroughly cleaned and activated to form a new oxide layer with the OH groups, three salinization methods with mixed SAM solution, APTES solution, and APS solution were used to perform the subsequent surface modifications.	For the immobilization of the IL-6 aptamer, the mixed SAMs solution was adopted for salinization process for the surface of the Si NWFET device. After the treatment of GA, the SiNW FET device was immersed in the aptamer solution to form covalent bond between the amine group at 5′ end of the aptamer and the aldehyde group on the device surface.	Silicon nanowire field-effect transistor (SiNW FET)	2.1 pg/mL (100 fM)	The lowest concentration of detection for the aptamer-functionalized SiNW FET was two orders of magnitude lower than the antibody-functionalized SiNW FET.	[176]
Troponin I (TnI)	The extended gate electrodes were fabricated with sequential deposition of metal layers using electron beam evaporation and the followed photolithography steps were performed to expose two specific regions on the gold electrodes: one served as the sensing electrode connected to the gate terminal of a metal–oxide–semiconductor field-effect transistor (MOSFET), while the other functioned as a reference electrode for applying the gate bias.	The aptamer was modified with a S-S bond at 5′ end, facilitating its anchoring to the Au electrode surface via thiol–Au chemistry. In the aptamer immobilization process, the aptamer was first combined with SDS to denature, followed by S-S reduction by TCEP to improve the aptamer immobilization onto Au electrode surface.	Extended-gate field-effect transistor (EGFET)	A linear response range of 1 ng/mL to 100 ng/mL	The use of interchangeable extended gate chips greatly improves the modularity and scalability of the system, making it suitable for healthcare applications.	[177]
Cardiac troponin I (cTnI, biomarker of acute myocardial infarction, AMI)	Constructed with ion-sensitive field-effect transistors (ISFETs) and an additional gate that connects with the gate of the ISFET and acts as the sensing area with ample space for molecule recognition and substrate exchange. A Prussian blue-gold nanoparticle (PB-AuNP) composite was immobilized on the extended gate of the EGISFET, providing rich active sites and excellent electrocatalytic ability to amplify signal for pH change. ISFETs have a dual gate working mode of a liquid gate coupling with a back gate where concentrations of targets are converted to pH changes during the detection.	Aptamer specific to cTnI was labelled with biotin at 5′ end was reacted with streptavidin and then with biotin-labelled HRP to prepare capture probe through the high affinity of a biotin-streptavidin-biotin structure. Afterward, auxiliary probe of complimentary strand DNA, thiolated at 3′ end, was dropped on the surface of the APTMS-modified EGISFET and covalently bonded with PB-AuNPs via Au–S bonding.	Extended-gate ion-sensitive field-effect transistor (EGISFET)	0.3 pg/mL with a linear range of 1–1000 pg/mL	A target-induced strand release strategy is demonstrated to shorten the recognition time of cTnI using a complimentary DNA strand for rapid detection and sensor regeneration. Potential for early warning and rapid diagnosis of AMI in emergency treatment.	[178]
Kanamycin (KAN)	The PEGFET sensor consists of an n-MOSFET, a TiO_2_/ITO extended gate electrode, and an Ag/AgCl reference electrode. The photoelectric gate electrode is made of ITO glass as the substrate, on which a photosensitive TiO_2_ layer (6 mm diameter circular hole) surrounded by a polyimide film works as the sensing area. The gate of the source metre is connected to an Ag/AgCl reference electrode, while its source and drain are connected to the source and drain of a commercial n-MOSFET. The gate terminal of the n-MOSFET is linked to a TiO2/ITO electrode, serving as an extended gate. Both the extended gate and Ag/AgCl reference electrode are immersed in the test solution to form a complete gate source circuit.	During the sensing process, the single-strand DNA (ssDNA) aptamer is linked onto the Au nanoclusters (NCs) for KAN recognition. When target KAN is present, the KAN molecules will bind to the surface of AuNCs and weaken their catalytic ability. Thus, the deposited DAB layer becomes thinner, and the photocurrent increases. Different amounts of DAB precipitate on the photoelectrode surface, leading to gate voltage shift and source-drain current response.	Photoelectrochemical extended-gate field-effect transistor (PEGFET)	At nM level with a linear range of 10 nM to 100 μM	Provide new insights for the development of EGFET sensor with photoelectrochemical process for practical sensing applications.	[179]
Brain-derived neurotrophic factor (BDNF)	Constructed on a flexible polyimide (PI) membrane coated with Au/reduced graphene oxide (r-GO) and utilized an extended-gate field-effect transistor (EGFET) as the transducer. The negatively charged DNA aptamers as recognition sites on the surface of the modified electrode enable the specific binding of BDNF within the r-GO/PBASE/APT/BSA modified electrode matrix. Applying a positive bias to the floating gate electrode attracts positively charged BDNF molecules at pH 7 to the modified electrode surface to enhance the binding and induce the surface potential charge for detection.	DNA aptamer specific to BDNF immobilized on sequentially modified PI membrane substrate with Au/r-GO/PBASE via a chemical linker EDC (1-ethyl-3-(dimethylaminopropyl) carbodiimide-NHS (N-hydroxysuccinimide) for coupling reaction with PBASE. The surface was further modified with BSA to enhance sensitivity and selectivity for BDNF.	Extended-gate field-effect transistor (EGFET)	0.4 nM with a dynamic range of 0.025 to 1000 nM	First electrical aptasensor for BDNF detection, reliable, highly sensitive, wide range, cost effective, fast response, flexible, portable, and early diagnosis.	[166]
Vascular endothelial growth factor 165 (VEGF_165_)	A DNA strand (T1) is to functionalize the **graphene** channel interface of gFET with PASE as the linker for capture of another DNA strand (T2) containing the sequence complementary to the aptamer (P1) specific to VEGF_165_, which was released from the double-stranded DNA-aptamer complex (P1-T2), owing to higher affinity of the aptamer P1 to VEGF_165_. The captured T2 could trigger hybridization chain reaction in the presence of hairpin H1 and H2 on the channel interface; thus, the DNA structure on the interface obviously changed in the presence of target protein. Due to being negatively charged, DNA hybridization enhanced the hole-doping effect on the graphene, which caused a target protein-related negative shift in Dirac voltage of the GFET device, and, thus, a sensitivity method for analysis of target protein could be realized by the DNA hybridization on the GFET-based biosensors.	T1 modified with amino group at 5′ end was covalently bound on the graphene interface using PASE as the linker while PASE could be absorbed on graphene *via π*–*π interaction*. P1 was the aptamer specific to VEGF165 and hybridized with T2. After the target VEGF_165_ was recognized by the aptamer, T2 could be released from P1-T2 complex to hybridize with T1 on the channel interface.	**Graphene** field-effect transistor (gFET)	3.24 pg/mL	The proposed biosensing strategy possessed excellent extensibility for other proteins or even nucleic acids by simply changing the specific aptamer.	[167]
Vascular endothelial growth factor (VEGF)	The remote dual gates were fabricated by coating poly [3-(3-carboxypropyl)thiophene-2,5-diyl] (PT-COOH) transducer film on SiO_2_/Si substrates. The probe functionalization was performed with aptamer DNA strand (VEap) either alone (PT-VEap) or in conjunction with its complementary single-stranded DNA (PT-VEap/CS). The remote dual gates were integrated with commercial MOS-FET for detection (as illustrated in Figure 10).	Amino-modified aptamer DNA strand VEap for VEGF121 were immobilized onto the PT-COOH film via acylation using EDC/NHS chemistry to form PT-VEap or PT-VEap/CS sensing surface on the remote gates. Charged backbones of the aptamers facilitated specific recognition in complex surroundings and maintained high sensitivity to VEGF across a broad pH range (5–9) and ionic strength (0.05–1.0 × PBS).	Remote dual-gate organic field-effect transistors (OFETs)	0.1 ng/mL	The PT-VEap and (PT-VEap/CS) aptamer sensors exhibited opposite changes in threshold voltage in response to VEGF, and the CS pairing helped PT-VEap/CS sensors achieve 10 times lower detection limit.	[168]
Escherichia coli (*E. coli*)	FET sensor was built based on rGO, gold nanoparticles (AuNPs), and an amino ssDNA aptamer to detect whole cells of *E. coli*. At first, source and drain electrodes were formed on silicon wafer using photolithography and lift-off process. Then, rGO was coated onto the sensor surface with GO solution via thermal reduction, followed by AuNPs sputtering.	Amine terminated amino ssDNA aptamer specific to *E. coli* was immobilized on the rGO surface by EDC/NHS chemistry.	rGO-AuNPs field-effect transistor	3 CFU/mL with a linear range of 3–3 × 10^6^ CFU/mL	Amine terminated ssDNA aptamers are less expensive than thiolated aptamers (attached to AuNPs) and provide a strong covalent bond with rGO; thus, FET performance was enhanced but its cost reduced.	[180]
Cortisol (a key stress biomarker)	To fabricate FETs on flexible substrates for conformal skin contact, thin-film In_2_O_3_ was formed on polyimide via spin coating In_2_O_3_ precursor followed by solution-processed sol-gel chemistry. The In_2_O_3_ layer was then patterned by photolithography and reactive ion etching to form the channel regions. Interdigitated Au/Ti electrodes were patterned to form source and drain contacts.	The In_2_O_3_ was functionalized with APTES and PTMS (1:9 *v*/*v* ratio) via self-assembly using vapour-phase deposition. Cortisol aptamer with a thiol modification at the 5′ end was covalently immobilized on amino-silanized In_2_O_3_ FET channels using MBS as a crosslinker.	Flexible field-effect transistor (FET)	1 pM with a dynamic range of 1 pM to 1 µM	Demonstrated the flexible FET array system can be straightforwardly adapted in wearable and mobile formats for additional physiological biomarkers, inc. targets at low concentrations in sweat.	[181]
Cortisol (a key depression marker)	ISEFT sensor was fabricated on n-type silicon wafer with multiple steps, including wet oxidation for SiO_2_ layer, boron implantation for contact interconnects, lithography lift-off process for source and drain contact patches with an Al/Ti/Au metal stack, another wet oxidation process to produce SiO_2_ layer to serve as a device passivation, and another lithography process to open window contacts for the source/drain/gate. Afterward, a dry oxidation process to grown thin-layer SiO_2_ to passivate the sensing area. Finally, another lift-off process to produce outer source/drain contacts using the similar Al/Ti/Au stack.	The ISFET surface was functionalized with APTES, self-assembled through vapour-phase deposition via salinization process. The APTES molecule features a group that covalently binds to gate oxide and an amine group at the other end, which can react further with linker MBS. The thiol group at the 5′ end of the aptamers cross-linked with the amine-terminated silanes via MBS.	Ion-sensitive field-effect transistor (ISFET)	1 fM with a dynamic range of 1 fM to 1 µM	Suitable for integration into portable and wearable devices. A great potential for the development of accurate point-of-care monitoring system for early detection of depression disorders.	[182]

### 3.4. Electrochemical Aptasensors

Electrochemical aptasensors use the electrode surface as a platform to immobilize the biosensing aptamer, convert the aptamer recognition event into an electrical signal, and detect changes in current, potential, or impedance to achieve the rapid detection of target molecules or target tissues [190,191]. The advantages of the electrochemical transduction mechanism are the simplicity of its conversion phenomenon and the possibility of using label-free and reusable detection systems. Electrochemical transduction is extremely sensitive and can be enhanced in practice by amplifying the detection signal by attaching a biocatalytic tag to the aptamer–target complex. In practical applications, electrochemical aptasensors are produced at a low cost and are universally applicable [192,193]. The following section will focus on electrochemical impedance spectroscopy (EIS) aptasensors, voltammetric aptasensors, and amperometric aptasensors.

#### 3.4.1. Electrochemical Impedance Spectroscopy Aptasensors

**Figure 12:**
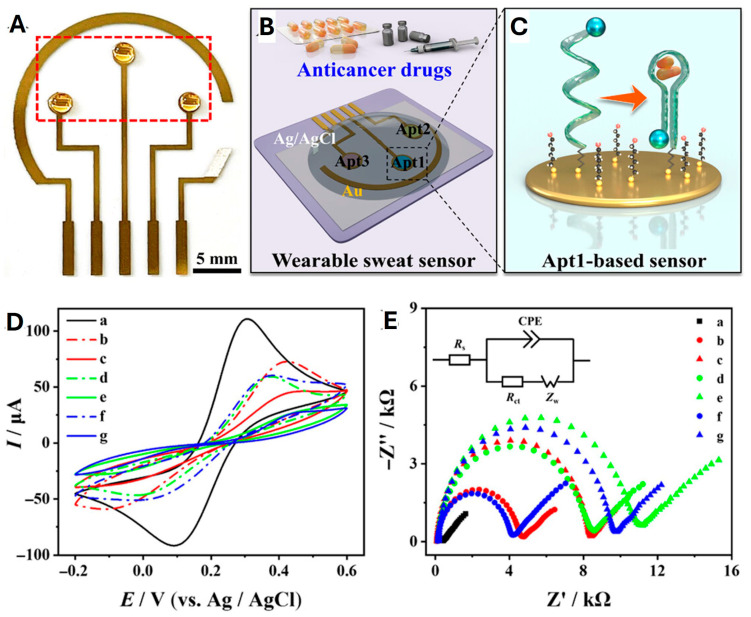
Integrated electrochemical aptasensor array for monitoring anticancer drugs in sweat. (**A**,**B**) are photograph and schematics of the integrated electrochemical sensor array for wearable sweat monitoring device. Three aptamers (Apts 1, 2, and 3), which are specific for anticancer drugs cyclophosphamide (CTX), cisplatin (DDP), and docetaxel (DTX), respectively, were modified with a thiol group at its 5′ end and methylene blue (MB) at its 3′ end. They were immobilized onto the surface of the Au working electrodes via S-Au covalent bond. (**C**) Zoomed-in schematic for the functionalized working electrode based on Apt1. When the target antidrug molecule in the testing solution is recognized and bound by the aptamer Apt1, the conformation of the aptamer is changed, and the molecular packing on the surface becomes denser or the distance between the electrochemically active MB and the electrode surface is reduced; thus, the electrochemical response (impedance, charge transfer, electron transfer, etc.) on the electrode surface changes. (**D**) Cyclic voltammograms and (E) Nyquist plots of EIS obtained at different modified electrodes: (a) bare Au, (b) Apt1/Au, (c) CTX/Apt1/Au, (d) Apt2/Au, (e) DDP/Apt2/Au, (f) Apt3/Au, and (g) DTX/Apt3/Au. The insert of (**E**) is the equivalent circuit for the impedance spectra. Cyclic voltammetry (CVs) and EIS were performed in 100 mM KCl containing 5 mM [Fe(CN)_6_]^3−/4−^. The concentrations of CTX, DDP, and DTX were 1, 50, and 0.5 μM, respectively. Reprinted with permission from Ref. [199]. Copyright 2024, American Chemical Society.

EIS is a label-free and non-destructive monitoring technique achieved by applying a small alternative current (AC) amplitude sinusoidal perturbation signal to the system in equilibrium [194,195]. EIS has an excellent performance in measuring the molecular interactions occurring at the electrode surface while not causing an electrochemical reaction under the applied AC signal, thus making it non-destructive [196]. It can detect any subtle changes at the interface induced by the interaction between the nucleic acid aptamer and the target molecule. EIS is commonly used to determine organic compounds, such as protein biomarkers, and can also be used to determine electrochemical changes occurring on the surface of modified electrodes due to biorecognition. Various biomolecules such as enzymes, antibodies, nucleic acids, and cells are immobilized on the surface of the electrodes and used as detection elements to develop EIS aptasensors [197,198]. Moreover, EIS aptasensors are inexpensive to manufacture and can be mass produced and reused, as demonstrated in Figure 12.

In EIS aptasensors, the total impedance can be divided into the real component and imaginary component, corresponding to the resistivity component and capacitance component, respectively. The resistivity component is caused by the obstruction of the electrode surface to the current flow, while the capacitance component reflects the storage of an electric charge in the system when the voltage is applied [200]. EIS applications in aptasensors can be classified into two types depending on the nature of the measured signal: Faraday and non-Faraday types. In Faraday-type EIS aptasensors, the electrode surface is partially or completely covered by a non-insulating layer or partially covered by an insulating layer. Impedance is generated when electrons are transferred to the electrodes through redox reactions. In non-Faraday-type EIS aptasensors, the electrode surface is completely covered by a dielectric layer, and the entire electrode assembly behaves as an insulator. The generation of impedance is based on alternative current impedance, at which point no charge is transferred to the electrode surface, but the current can still flow, so the system behaves like a capacitor [201,202]. Therefore, the non-Faraday-type EIS aptasensors are also known as the capacitive EIS aptasensors. Faraday-type EIS aptasensors are generally considered to have a higher sensitivity than capacitive ones, so they can be used for the determination of target molecules rather than simply detecting their presence [203]. Moreover, Faraday-type EIS aptasensors can produce a more pronounced signal change at the same concentration of the target, thus having a better sensitivity.

Unlike other conventional biosensors, the EIS aptasensors directly use aptamers as the core recognition element, which encounters the electrolyte. Therefore, if EIS aptasensors are to be more widely applied in other fields, the aptamer-based electrodes selected must have long-term stability when being in contact with any electrolytes. Compared with other biosensors, EIS aptasensors have a better stability, higher sensitivity, and are applicable to multiple media rather than a single medium, which require complex and efficient electrode designs to immobilize the aptamer on the electrode surface [204]. This is to ensure that the aptamer and the electrode can be effectively connected, utilizing the characteristics of aptamers as recognition elements, which can generate stable signals for detection purposes. The strategies for the better construction and optimization of EIS aptasensors is also one of the main research directions in future.

#### 3.4.2. Voltametric Aptasensors

In addition to EIS, voltammetry is also used for electrochemical aptasensors. Voltammetry is the oldest and one of the most commonly used electrochemical techniques. The voltametric transduction mechanism involves scanning the potential on the working electrode from one preset value to another and recording the electrochemical current as a function of the applied potential [205]. The use of voltammetry for electrochemical aptasensors has been made more widespread with the continuous advancement of computerized techniques for controlling and measuring potentials and currents in constant potentiostats [206]. Voltametric aptasensors are widely used for the detection of protein biomarkers because of their extreme sensitivity and the ease with which the experiments can be performed and reproduced [207].

Voltametric aptasensors obtain information about target molecules by measuring the changes in current signals within a variable potential range and simultaneously detect changes in potentials and currents [208,209]. Voltammetry methods applied in aptasensors can be classified as cyclic voltammetry (CV), differential pulse voltammetry (DPV), square wave voltammetry (SWV), alternating current voltammetry (ACV), and linear sweep voltammetry (LSV), based on different technical principles [210]. These different voltametric techniques, when combined with aptasensors, can detect various types of target molecules, thereby increasing the selectivity and application range of voltametric aptasensors. Voltammetry generally uses a standard three-electrode system (working electrode, counter electrode, and reference electrode) by applying a certain form of electrical potential that triggers the redox reaction of the electroactive substance on the working electrode and then records the current changes within a specified time range. The voltametric method reflects the electrochemical characteristics of target molecules from different angles through potential, current, and time functions. The voltammogram records the current curves of the oxidation and reduction processes of the detected target substance with the electroactive substances in the working electrode at a specific potential. In the voltammogram, data such as the peak value, peak potential, and peak width of the curve reflect the electrochemical characteristics of the target molecules or target substances.

Voltametric aptasensors have low noise characteristics during operation and can detect multiple target substances with different peak potentials in a single scan. Voltametric aptasensors can also characterize multiple target molecules as a whole and can be integrated into the sensing system to form a voltametric detection system [211], as demonstrated in Figure 12D. Even in the presence of some interfering substances, the voltametric aptasensors can still detect single or multiple groups of target substances simultaneously. In voltametric aptasensors, the limit current is affected by the testing temperature, so it should be kept at a constant temperature during detection to ensure the accuracy of the results obtained [212]. Voltametric aptasensors have a great commercial potential due to their low manufacturing cost in addition to their high sensitivity.

#### 3.4.3. Amperometric Aptasensors

Amperometric detection is another commonly used transduction mechanism for electrochemical aptasensors. The amperometric aptasensors are relatively self-contained electrochemical devices. They work by converting the biorecognition changes that occur as a result of oxidation or reduction in an electroactive substance into a current signal, which is subsequently detected and quantitatively analyzed [213].

Amperometric aptasensors obtain information about target molecules by measuring the changes in current signals at a constant potential. The magnitude of the current generated by oxidation or reduction reactions can enable accurate quantitative analysis by the amperometric aptasensors. The main difference between amperometric and voltametric aptasensors is that amperometric aptasensors applied a constant potential. In amperometric aptasensors, the potential is directly adjusted to the specific value and maintained, only allowing the target substance to be reacted on the surface of the electrodes, after which the output current is recorded and used for detection and analysis. The higher output current of the amperometric aptasensors represents the higher concentration of the electroactive substance in the target substance sample being detected [214,215,216].

One of the most crucial aspects in the development of amperometric aptasensors is the selection of the strategies to improve their selectivity, sensitivity, and detection range. For example, to address issues with the disparity of the concentrations of the targets existed in different conditions, great efforts have been made to optimize the designs of amperometric aptasensors. It has been observed that the affinity of the aptamers has an influence on the detection range of the amperometric aptasensors and that the aptamers with a stronger affinity possess a larger detection range than those with weaker affinity [217]. Therefore, improving the affinity of the aptamer is proposed to be an effective approach to extend the detection range of the amperometric aptasensors. Aptamer affinity can be improved for amperometric aptasensors through sequence optimization, aptamer structure stabilization, and the introduction of hydrophobic groups [218]. In addition, a multi-aptamer strategy can also be adopted by incorporating two or more aptamers with different affinities to simultaneously measuring the concentrations of different target substances for the purpose of expanding the detection range [219]. The properties of the linkers between the aptamer and the electrode also influence the detection of the amperometric aptasensors, affecting the sensitivity and detection range of the amperometric aptasensors. Generally, the rigid linkers used as flexible linkers can comprise the sensing performance of amperometric aptasensors under certain circumstances [220]. However, when a rigid linker and a flexible linker are simultaneously introduced into multi-aptamer amperometric aptasensors, it is interestingly noticed that the multi-aptamer sensors exhibited a higher sensitivity, greater selectivity, and broader detection range than either the single-aptamer amperometric aptasensors or multi-aptamer sensors using a single linker approach [221]. In addition, a passivation layer can be added to the electrode surface to suppress background noise from the electrode itself [222].

## 4. Post-SELEX Optimization Process of Aptamers

**Figure 13:**
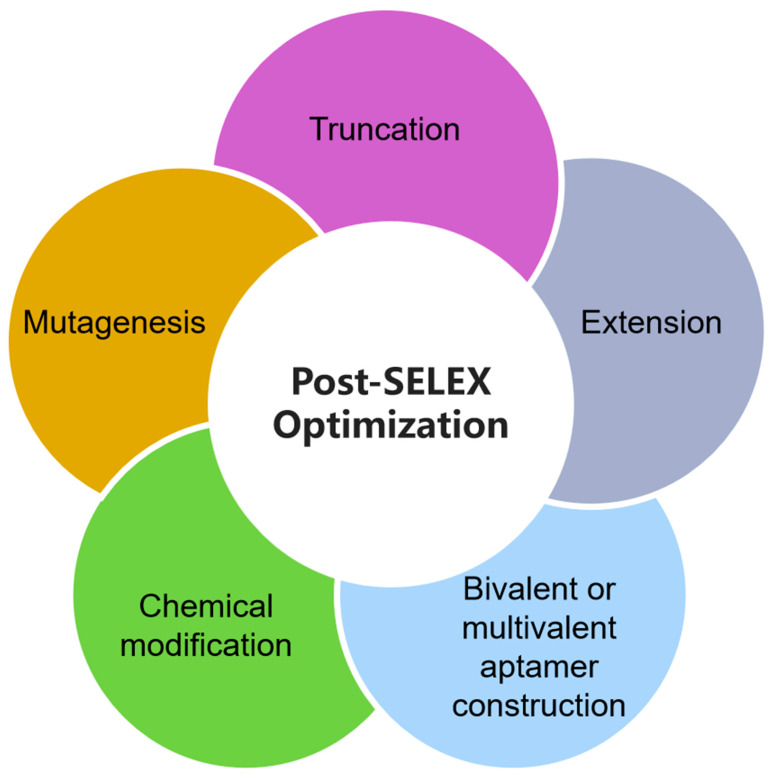
Commonly used post-SELEX optimization methods.

In recent years, there has been a rapid development of nucleic acid aptamers, which has led to a wide range of applications in a variety of fields such as biosensors, medical diagnostics, food safety, and environmental monitoring [223,224,225,226,227,228,229]. However, the overall performance of nucleic acid aptamers screened by SELEX still needs further improvement. One of the key reasons for the limited performance of nucleic acid aptamers is their poor stability and reproducibility [230]. Nucleic acid aptamers screened by various SELEX technologies have drawbacks that limit their use in target molecule monitoring under complicated conditions [231]. In addition, for real-world applications, nucleic acid aptamers have to fulfil other requirements such as reduced production costs, integration with existing manufacturing processes, and sufficient bioavailability [232]. In order to achieve performance improvement and a cost reduction in nucleic acid aptamer applications, a variety of post-SELEX optimization methods have been developed. As shown in Figure 13, the commonly used post-SELEX optimization methods include truncation, mutagenesis, extension, chemical modification, and bivalent or multivalent aptamer construction.

In 2009, Chushak and Stone [233] proposed a computational approach for designing a starting pool of RNA sequences for the selection of RNA aptamers for specific analyte binding. The approach consisted of three steps: (i) selection of RNA sequences based on their secondary structure, (ii) generating a library of three-dimensional (3D) structures of RNA molecules, and (iii) high-throughput virtual screening of this library to select aptamers with a binding affinity to a desired small molecule. A set of criteria were also set up to rank and filter the top sequence candidates to be verified. The proposed approach reduced the RNA sequences search space by four to five orders of magnitude, significantly accelerating the experimental screening and selection of high-affinity aptamers. Complementary to SELEX, the in silico aptamer selection and design strategy has been extensively investigated to successfully facilitate the virtual screening and increased understanding of important nucleic acid–protein interactions. For example, Soon and Nordin [234] used computational molecular docking and the three-dimensional structure information of nucleic acid sequences to achieve in silico refinement of the desired binding requirement on the target Streptococcus agalactiae. Bavi et al. [235] reported an in silico computational approach for the selection of aptamers for proteins, which involves the generation of a virtual library of sequences, modelling of their 3D-structures, and the selection of perspective aptamers through docking, molecular dynamics simulation, binding-free energy calculations, and, finally, estimating the experimental affinity. Using this method, a 15-mer RNA aptamer was successfully designed for an epithelial cell adhesion molecule. Bell et al. combined an experimental and theoretical approach to develop two optimal epithelial cellular adhesion molecule (EpCAM) aptamers. Their structure-based in silico method first predicted their binding modes and then optimized them for EpCAM with molecular dynamics simulations, docking, and free energy calculations. Nevertheless, the in silico method proved useful to gain mechanistic insights (binding conformation, contacts, and energetics) for the rational design of modifications and structure-based optimization or when the 3D structure of the target is well resolved via crystallography, cryo-EM, or AlphaFold. There are some limitations associated with structure-based in silico aptamer selection and design due to less reliable RNA/DNA folding predictions, noisy docking scores, and expensive computation (especially for molecular dynamics simulations).

With the advances of machine learning and AI tools, predictive models and computational tools are also being adopted for aptamer screening. These predictive models and computational tools incorporating machine learning and deep learning are essential in the post-SELEX optimization process to simulate the affinity of a nucleic acid aptamer for a target molecule, to simulate the dynamic binding process, and to determine the minimum sequence that binds to the target with high affinity and specificity [236,237,238,239]. In this section, we will focus on truncation methods and their associated prediction algorithms and computational tools.

### 4.1. Truncation

The truncation method is based on the principle of partial nucleotide removal to improve the aptamer performance and reduce manufacturing costs. Nucleic acid aptamers screened by SELEX typically consist of a random region of 30 to 50 nucleotides with constant primer sequences on both ends and are used for PCR amplification [240]. However, not all nucleotides will interact directly with the target molecule or the target substance, and those that are not necessary will instead lead to a more efficient and less costly synthesis of nucleic acid aptamers. Typically, the key nucleotides of the constant primer sequence and the aptamer-binding site are retained, and nucleotides outside the constant primer sequence and the aptamer-binding site are truncated.

The structure of the nucleic acid aptamer has a great impact on the effectiveness of the truncation technique, so if the secondary structure of the nucleic acid aptamer can be predicted, it can make the truncation optimization more effective [241,242]. Computational tools such as Mfold, NUPACK, and RNAstructure have been developed to predict the secondary structure of nucleic acid aptamers [243,244,245]. After the secondary structure of the nucleic acid aptamer is predicted by these computational tools, the information on the secondary structure is used to perform truncation optimization, thus maximizing the nucleotide length to improve the affinity of the nucleic acid aptamer. In a subsequent study, the structures of the aptamers are analyzed using Mfold, and the truncation is optimized based on the predicted structures, ultimately screening three aptamers from cancer cells. Upon further analysis, it was found that several of the aptamer primer sequences obtained were structurally similar and that the truncated primer sequences had a greater affinity than the original primer sequences [246]. In 2017, stepping libraries were created, and SPR was used as an effective screening and evaluation method to obtain the minimized aptamer In27 with only 35 sequences, significantly shortened from typical aptamers of about 70–130 nucleotides. The aptamer In27 has only one loop but retains the full binding affinity, which demonstrates that efficient post-SELEX truncation strategies have been successfully adopted [247]. A novel aptamer truncation strategy has also been demonstrated using molecular docking technology, which helped successfully obtain aptamer primer sequences that bind to the target molecules with increased affinities [248].

### 4.2. Other Post-SELEX Optimization Methods

The principle of mutagenesis starts from the existing aptamer primer sequences and uses predictive models and computational tools to accelerate aptamer optimization through modifying nucleotides at key positions [40,249]. Site-directed mutagenesis is the process of changing the nucleotide composition of an aptamer by mutating single or multiple nucleotides to obtain an optimized aptamer with better specificity as well as affinity for the target molecule. The mutagenesis method can also change the spatial structure of the aptamer to achieve an enhanced performance [250]. The extension method is to extend the aptamer primer sequence to introduce some nucleotide fragments with special structures into the aptamer to improve its performance. This method enhances the originally weaker properties of the aptamer and turns the disadvantage of the original aptamer into an advantage, allowing the aptamer to be used in a wider range of applications [251,252]. Chemical modification technology is used to modify nucleotides by adding chemical groups to enhance the interaction between the aptamer and the target molecule and to improve the properties of the aptamer. Chemical modifications can be classified as modifications of the sugar ring, modifications of the bases, and modifications of the linkage [253]. Bivalent or multivalent aptamer construction methods combine multiple aptamers through covalent bonds to overcome the short retention time and lack of crosslinking of monovalent aptamers on target molecules [254]. Multivalent aptamers have many advantages over monovalent aptamers, such as a higher sensitivity, higher conformational stability, and higher binding affinity [255,256,257]. In addition, multivalent aptamer construction methods can link different kinds of aptamers together, even if these aptamers recognize different target molecules or target substances [258,259,260]. The multivalent aptamers constructed by this method can recognize target substances containing different ligands and can also recognize multiple different target substances simultaneously [261,262,263,264].

## 5. Conclusions and Outlook

This review provides a detailed description of nucleic acid aptamers and SELEX technologies for the in vitro screening of nucleic acid aptamers, and it also debriefed the principles and advantages and disadvantages of three main SELEX technologies developed based on the separation and partition of target-bound sequences from non-binding ones. In addition, the working principle and design concept of aptasensors are also summarized with the focus on the advances and challenges of three important aptasensors, namely optical aptasensors, field-effect transistor (FET) aptasensors and electrochemical aptasensors. Finally, this review provided insights for post-SELEX optimization methods that combine advanced prediction algorithms and computational tools with efficient SELEX techniques for practical applications, describing their methodological principles and technical features.

Great efforts on nucleic acid aptamer research are continuing, although SELEX technologies and their optimization methods have been significantly advanced. With the advent of machine learning and AI technology, predictive models and computational tools will be further adopted for shortening aptamer discovery while simultaneously enhancing their affinity and specificity. With the increasing specificity and sensitivity of nucleic acid aptamers and the improvement of SELEX technology, low-cost and high-performance aptasensors can be achieved for medical diagnostics, environmental monitoring, and food safety applications. The research of nucleic acid aptamers and aptasensors is still of great significance in advancing the development of innovative diagnostic tools in nucleic acid aptamer-based bioassays.

Although remarkable achievements have been made in nucleic acid aptamers and aptasensors, some limitations and great challenges are to be addressed to fully exploit their potential in a wide range of real-world applications.

(1) It is more difficult to screen aptamers for small molecule targets than for large ones. It is worth highlighting a new type of SELEX technology, named Capture-SELEX, can be used in the field of screening aptamers for small molecule targets, despite that few SELEX technologies applied in this field [265].
(2) Point-of-care (POC) diagnostic systems have become increasingly demanded in healthcare and clinical diagnosis; aptamer-based biosensing systems have proven their feasibility, but they are still in their infancy. There is still a significant gap in affordability, standardization, and commercialization [266,267,268].
(3) Wearable aptasensors are a brand-new field that combines flexible materials, artificial intelligence, machine learning, and aptasensors, but it is still in its infancy at present. In the future, there are still huge challenges ahead in improving the consumption of wearable devices, the collection of detection data, and the storage of wearable aptasensors under various physiological conditions and in complex external environments [269].
(4) Besides aptamers, some emerging nanomaterials also demonstrated promise for the enhanced performance of aptasensors, despite some condition limitations observed [270,271]. For example, the sustainability and toxicity of nanomaterials in sensor applications have been insufficiently investigated. The fabrication of nanomaterials and nano-biosensors is usually complicated and investment-intensive, which could also cause affordability and accessibility issues for nanomaterial-based aptasensors. Thus, more research should be performed to address these issues.
(5) The discovery of aptamers and their applications in sensing have become an interdisciplinary research field across physics, chemistry, biology, materials science, and computer science, and several recent aptasensor designs have demonstrated that deep learning and predictive models can effectively enhance the performance of aptasensors, while significantly shortening the discovery time of the aptamers as well as running costs [272,273,274,275,276,277,278,279,280,281,282,283,284,285]. In the future, the development and adoption of advanced predictive algorithms and computational tools are expected to have a significant impact on the development of high-performance and low-cost aptasensors. Especially, hybrid in silico approaches combining machine learning (ML) and structure-driven modelling will be increasingly common for the balanced benefits in scalability (ML) and physical realism (structural modelling).

In conclusion, as previously mentioned, there are still some great challenges for the wide exploitation of nucleic acid aptamers for sensing applications. With the aid of emerging machine learning and AI tools, better understanding of aptamer–molecule target interactions and configuration changes in real time would enable us to fully explore nucleic acid aptamers and aptasensors.

## Figures and Tables

**Table 1:** Summary of the advantages and limitations of aptamers compared with antibodies.

Feature	Aptamers	Antibodies
**Nature**	Short ssDNA or RNA oligonucleotides	Large protein molecules (~150 kDa)
**Production**	Fully synthetic via SELEX	Biological (immunization, hybridoma, and cell culture)
**Time to Develop**	Weeks	Months
**Batch Consistency**	High (chemical synthesis)	Variable (biological expression)
**Size**	Small (5–15 kDa)	Large (~150 kDa)
**Target Range**	Proteins, small molecules, toxins, ions, and even non-immunogenic targets	Mostly proteins and larger antigens
**Stability**	Stable to pH, heat; reversible folding	Sensitive to temperature, pH; irreversible denaturation
**Modification**	Easily and precisely modified (labels, drugs, and nanomaterials)	Modifications more limited and complex
**Tissue Penetration**	Better (small size)	Limited (large size)
**Immunogenicity**	Very low	May trigger immune responses
**Cost**	Relatively low (chemical synthesis)	Higher (animal/cell-based production)
**Regulatory Approval**	Fewer approved (e.g., Pegaptanib)	Many approved therapeutics and diagnostics
**Degradation**	Susceptible to nucleases (can be stabilized chemically)	Proteolytic degradation but generally longer half-life in vivo

## Data Availability

No new data were created or analyzed in this study.
